# Alkali Ions Retained from Synthesis Govern Microstructure and Transport in Solution-Processed SnSe

**DOI:** 10.1021/acs.chemmater.6c01284

**Published:** 2026-09-08

**Authors:** Christine Fiedler, Sharona Horta, Navita Jakhar, Krishnendu Maji, Tobias Kleinhanns, Jordi Llorca, Qi Wang, Maria Garcia-Ramon, Sambit Das, Vikram Gavini, Yu Liu, Maria Ibáñez

**Affiliations:** † 148492 Institute of Science and Technology Austria (ISTA), 3400 Klosterneuburg, Austria; ‡ Department of Chemical Engineering and Barcelona Research Center in Multiscale Science and Engineering, 16767 Universitat Politècnica de Catalunya, EEBE, Av. Eduard Maristany 10-14, Barcelona 08019 Catalonia, Spain; § 1259 University of Michigan, 500 S State Street, Ann Arbor, Michigan 48109, United States; ∥ Anhui Province Engineering Research Center of Flexible and Intelligent Materials, School of Chemistry and Chemical Engineering, 558979 Hefei University of Technology, 230009 Hefei, China

## Abstract

Alkali salts are widely used in solution processing, yet the corresponding cations are often assumed to remain in the liquid phase. Here, we show that alkali ions introduced during the solution synthesis of SnSe are retained through particle isolation and continue to influence the material during subsequent thermal processing, where they shape the evolution of the final polycrystalline material. Different SnSe powders were prepared using Li^+^, Na^+^, and K^+^ precursors, as well as tetramethylammonium (TMA^+^) precursors as a nonalkali reference for comparison, and all samples were consolidated under identical conditions. Although all three alkali ions were found in the matrix, at grain boundaries, and in segregated nanoscale regions, they partitioned differently and resulted in distinct grain sizes, defect distributions, and therefore transport properties. Li induced the strongest grain size heterogeneity and the lowest energy barriers, Na the best overall balance between carrier concentration and mobility, and K the broadest alkali-rich grain boundary regions along with the strongest interfacial penalty to charge carrier transport. These results show that residual alkali ions are not just remnants of solution synthesis but active determinants of microstructure and thermoelectric performance in SnSe.

## Graphical abstract

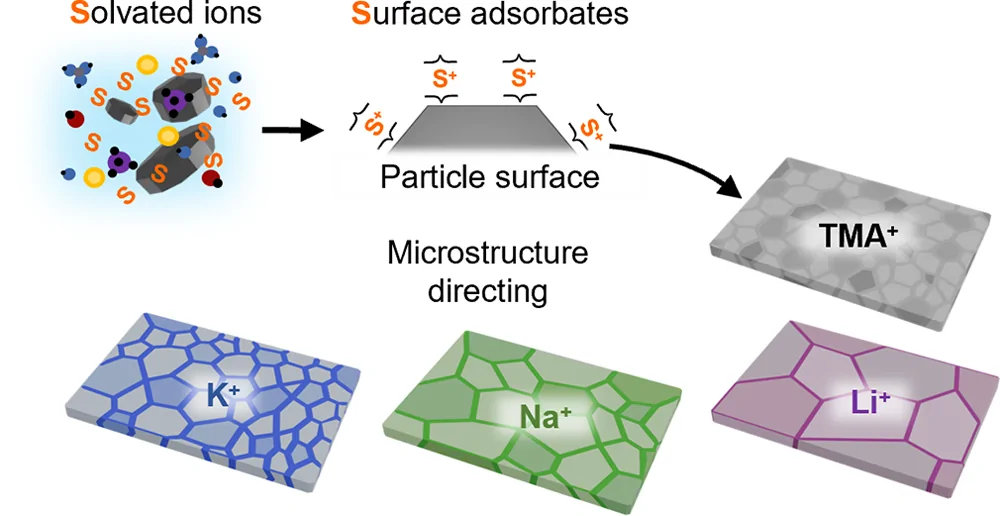

## Introduction

In the solution-based synthesis of inorganic particles, alkali salts are commonly used to regulate precursor chemistry,^1,2^ redox processes,^3–5^ and particle formation,^6–12^ where alkali ions balance charged species and that influence the ionic environment during nucleation and growth.^11,13,14^ In many cases, however, the resulting particles are not the final product but precursors whose composition and surface chemistry influence their subsequent conversion into functional materials, including dense polycrystalline solids,^15–21^ thin films,^22^ solar cell absorbers,^23^ catalysts,^24^ or battery electrodes.^24,25^ This progression from solution-based synthesis to functional solids means that alkali ions introduced to direct solution chemistry cannot always be regarded as species confined to the liquid phase, as they may remain associated with the isolated particles and continue to influence the material during later processing.

The retention of alkali ions is especially important when the final structure and properties emerge during annealing, consolidation, or device fabrication rather than during particle formation itself.^26–30^ Residual alkali ions carried by the isolated powders can then affect defect formation,^15,31^ crystallization pathways,^32^ grain boundary chemistry,^33^ and phase evolution,^34^ even when they are not readily detected by routine characterization.^35–38^ In our previous work, we established this point for solution-synthesized SnSe powders prepared in the presence of Na-containing reagents, showing that Na^+^ remained associated with the particle surfaces and subsequently altered the microstructure and transport properties of the consolidated material.^31^ That study, however, examined a single cation, Na^+^, and established only that retained alkali ions can influence the final microstructure. It did not address whether cation identity leads to distinct outcomes across alkali ions due to the differences in ionic size or side preference in the material.

SnSe is an excellent model system to address this question because its electrical and thermal transport properties are strongly affected by both defect chemistry and microstructure.^15,39,40^ Here, we compare SnSe powders synthesized in the presence of Li^+^, Na^+^, and K^+^, with tetramethylammonium (TMA^+^) as a nonalkali reference, and consolidate all powders under identical annealing and SPS conditions. This comparison allows us to determine how the identity of the alkali ion influences the development of the grain morphology, defect distribution, and thermoelectric transport properties of the final material.

## Experimental Section

### SnSe Particle Synthesis with Alkali Bases

SnSe particles were prepared following a procedure previously reported by D. H. Gregory et al.,^41^ with slight modifications. In a typical synthesis, NaBH_4_ (160 mmol) was first dissolved in 200 mL of deionized water, after which Se powder (80 mmol) was slowly added to the solution. Stirring was avoided at this stage because hydrogen gas was released during the formation of NaHSe. An additional 200 mL of Milli-Q water was then added. Once the bubbling had ceased, stirring was resumed, and an Ar flow was introduced until the solution became transparent, indicating the complete reduction of Se. In parallel, NaOH (750 mmol) and SnCl_2_·2H_2_O (72 mmol) were mixed with 360 mL of deionized water. The mixture was stirred at room temperature under Ar flow until complete dissolution was achieved. The solution was then heated under reflux to its boiling point (ca. 101 °C). The freshly prepared selenide solution was rapidly injected (11 mL/s) into the boiling Sn^2+^ solution, causing the temperature to drop to ∼70 °C. Upon injection, the solution turned black, indicating nanoparticle formation. The temperature was allowed to recover to ∼101 °C and was maintained at this value for 2 h.

For the synthesis of the other particles (TMA-SnSe, Li-SnSe, and K-SnSe), the same procedure was followed using equivalent amounts of TMABH_4_, LiBH_4_, and KBH_4_ for Se dissolution and TMAOH, LiOH, or KOH for Sn dissolution. Because LiBH_4_ reacts readily with water, the water was first cooled to ca. 5 °C using an ice bath before the addition of the precursor. The solution was then allowed to warm slowly to room temperature before the addition of elemental selenium. The remainder of the synthesis was carried out in the same manner as for the other alkali salts.

### Purification of all SnSe Particles

To purify the as-synthesized particles, magnetic stirring was first stopped for ∼5 min, and the transparent supernatant was carefully removed. The remaining crude solution (ca. 160 mL) was transferred into four centrifuge tubes. The crude solutions were centrifuged at 6000 rpm for 1 min to isolate the particles, after which the supernatant was discarded and the purification procedure was initiated. The particles were purified by three precipitation/redispersion cycles using deionized water and ethanol alternatively. In all steps, the dispersions were vortexed for 1 min, sonicated for 5 min, and vortexed again for an additional 1 min before centrifugation. In the first cycle, fresh deionized water (total volume: 160 mL) was added to the particles, followed by centrifugation at 6000 rpm for 1 min. The supernatant was discarded, and ethanol (total volume: 140 mL) was then used to redisperse the particles, followed by centrifugation at 8000 rpm for 5 min. In the second cycle, deionized water was added to solubilize the remaining impurities, and particles were precipitated by centrifugation at 9000 rpm for 5 min. Afterward, ethanol was employed to redisperse and precipitate the particles by centrifugation at 8000 rpm for 5 min. The third cycle repeated the same procedure as the second cycle. Finally, the washed particles were dried under vacuum (<5 mbar) overnight at room temperature and kept in a N_2_-filled glovebox for further use.^42^

#### Powder Annealing

All dried SnSe powders were annealed at 500 °C under a forming gas (5%H_2_ + 95%N_2_) flow for 1 h inside a tubular furnace with a heating rate of around 10 °C min^–1^.

#### Pellet Consolidation

The annealed powders were ground into a fine powder with an agate mortar and loaded into a graphite die in the glovebox before being pressed into cylinders (Ø 8.6 mm × 12 mm). The sintering process was carried out under vacuum, in an AGUS PECS Spark Plasma Sintering (SPS) System-Model SPS 210Sx, to simultaneously apply a pressure of 47 MPa at a temperature of 500 °C for 5 min. The relative densities of the compacted pellets were measured by Archimedes’ method and were all above 92% of the theoretical value in all samples. All sintered cylindrical pellets were annealed under a forming gas (95%N_2_ + 5%H_2_) atmosphere (closed system) from room temperature to 500 °C at a rate of 10 °C min^–1^ and then maintained at the set temperature (500 °C) for 1 h before transport measurements. From these cylinders, the round shape pellets and rectangular bars were cut obtaining two normal directions, i.e., along the pressing direction and within the cylinder plane.

#### Structural Characterization

##### XRD Analysis

X-ray diffraction (XRD, 2θ angle: 20° to 60°; scanning rate: 5°/min) analyses were carried out on a Bruker AXS D8 ADVANCE X-ray diffractometer with Cu–Kα radiation (λ = 1.5406 Å). The size and morphology of initial particles, annealed powders, and sintered pellets were examined by field-emission scanning electron microscopy (SEM) on an Auriga Zeiss operated at 3.0 kV.

The pellets were analyzed also by the following method.

##### Synchrotron XRD

Experiment: Synchrotron XRD was conducted at DESY beamline P02.1 using high-energy synchrotron radiation (∼60 keV) with X-ray wavelength of 0.207377 Å. Experiments were performed on ground pellet powders by filling them in quartz capillaries with 2-theta spacing of 0.05 degrees. A total of 1 min scans of diffraction data were collected. Rietveld refinement was done by using FullProf software.

#### TEM Sample Preparation and Characterization

For microstructural characterization, transmission electron microscopy (TEM) lamellae were prepared by using a Thermo Fisher Scientific Aquilos 2 Cryo-FIB/SEM system. Electron-transparent lamellae were fabricated following the site-specific preparation protocol described by Schaffer et al.,^43^ which enables high-quality cross-sectional specimens with minimal damage. Final thinning of the lamellae was conducted using low-energy ion milling to reduce surface amorphization and minimize beam-induced artifacts introduced during the earlier stages of focused-ion-beam milling. Following preparation, the specimens were transferred to the TEM system using a vacuum transfer system to avoid exposure to air and prevent oxidation or other forms of atmospheric contamination. TEM imaging and energy-dispersive X-ray spectroscopy (EDX) data were acquired by using a JEOL JEM-2800 transmission electron microscope operated at an accelerating voltage of 200 kV. The collected data were subsequently processed and analyzed by using DigitalMicrograph software.

#### EBSD Characterization

To estimate the grain size, electron backscatter diffraction (EBSD) mapping was performed by using an FEI Helios PFIB Dual Beam FIB-SEM system. Sample preparation for EBSD was carried out using the rocking polishing method, as described by Zaefferer and Elhami.^44^ This method helps minimize the surface deformation and is effective in obtaining the high-quality surfaces for EBSD analysis. Initial surface polishing was carried out using a focused ion beam at 30 keV with a beam current of 1 nA to remove the rough surface of the pellet. This was followed by a final cleaning step at a reduced current of 0.3 nA to minimize surface redeposition and enhance the pattern quality.

EBSD data acquisition was performed using an Oxford Instruments NordlysNano detector in conjunction with Aztec analysis software. The acquired orientation maps were subsequently used for quantitative grain size analysis. The weighted average fraction was used to determine the size of the grains; due to the abnormal size distribution of the grains, of which number-based averages can obscure this behavior due to the overwhelming presence of smaller grains, area-weighted calculations better reflect the true microstructural impact of the abnormally large grains, which occupy a disproportionate fraction of the total grain area. By weighting grain size by area, this method captures the influence of these dominant grains on the bulk properties and provides a more representative metric of microstructural evolution.

#### APT Characterization

APT experiments were conducted to investigate the composition of the SnSe samples doped with Li. The sample preparation procedure used for APT needle-like specimens was performed using a Xe plasma FIB lift-out and milling on the Thermo Fisher Helios PFIB-SEM system. APT experiments were operated on the CAMECA LEAP 6000 XR instrument using laser pulsing mode at 5 pJ laser energy, under *a* < 10–10 Torr vacuum. All the tips were measured at 40 K, with a detection rate of 5 ions per 1000 pulses, a pulse rate of 67 kHz, and a pulse fraction of 20%. APT reconstruction and analysis were carried out using the AP Suite 6.3 software.

#### Density Functional Theory Calculations

All DFT calculations were conducted using the QUANTUM ESPRESSO software^45^ with PBE exchange–correlation functional and ONCV pseudopotentials from the pseudodojo database.^46^

#### Measurement of the Thermoelectric Properties

Seebeck coefficients were measured by using a static DC method. Electrical resistivity data were obtained by a standard four-probe method. Both the Seebeck coefficient and the electrical resistivity were measured simultaneously in an LSR-3 LINSEIS system in the temperature range between room temperature and 823 K, under helium atmosphere. At each temperature, 3 heating and cooling measurements were taken. Considering the system accuracy and the measurement precision, we estimate an error of ca. 4% in the measurement of the electrical conductivity and Seebeck coefficient, respectively. The thermal conductivity was calculated by κ_tot_= λ*C*_*p*_ρ, where λ is the thermal diffusivity, *C*_*p*_ is the heat capacity, and ρ is the mass density of the specimen. An LFA 1000 Laser Flash was used to determine λ of the samples with an estimated error of ca. 2.4%. The constant pressure *C*_*p*_ was estimated from the empirical formula by the Dulong–Petit limit (3R law), and ρ values were measured using the Archimedes method. To avoid cluttering of the plots, error bars were not included in the figures. The Hall coefficient was measured using the AC method at 5 mA and −0.7–0.7 T using HCS 10 (Linseis), from which the Hall carrier concentration and Hall carrier mobility were obtained.

## Results and Discussion

SnSe particles were synthesized through an aqueous chemical route in which tin and selenium precursors react in the presence of alkali salts. The alkali salts serve two roles during synthesis. First, the alkali hydroxides (LiOH, NaOH and KOH) establish the alkaline conditions required to stabilize the reactive tin species, within the basic range that supports the formation of Sn-hydroxide complexes that give rise to SnSe particles.^42^ Second, an alkaline, reducing environment maintained by the respective alkali borohydrides (LiBH_4_, NaBH_4_, and KBH_4_) enables the dissolution of elemental selenium and generates soluble reduced selenide species.^42,47^ Under these basic conditions, the alkali cations (Li^+^, Na^+^, or K^+^) act mainly as counterions, balancing charge and contributing to the stabilization of soluble precursor species. Upon mixing the Se and Sn precursor solutions, SnSe nuclei form and subsequently grow into crystalline particles. During this process, alkali ions can accumulate near the particle’s surface through electrostatic interactions, influencing surface charge compensation, growth, and aggregation.^15,16,31,42^ To contrast the behavior of inorganic alkali cations with that of an organic cation that does not introduce a persistent inorganic cation into SnSe, we also prepared reference samples using tetramethylammonium (TMA^+^) salts. The use of TMAOH and TMABH_4_ establishes the same alkaline and reducing conditions as the alkali-based reagents, and TMA^+^ can also associate with the particle surface during synthesis. However, unlike Li^+^, Na^+^, or K^+^, TMA^+^ predominantly decomposes into volatile fragments during the high-temperature annealing step. APT analysis confirms only minor traces of N and C in the final pellet, with limited aggregation at grain boundaries (Figure S1), at a much lower level than the pronounced alkali segregation observed in alkali-containing samples.^31^ TMA is therefore used here as an alkali-free reference, allowing us to isolate the effects specific to alkali cation identity. For clarity, we adopt the following nomenclature to distinguish the samples: TMA-SnSe, Li-SnSe, Na-SnSe, and K-SnSe.

All syntheses yield polydisperse SnSe particles (Figure S2), with negative zeta potentials (TMA-SnSe; −23 ± 5 mV, Li-SnSe; −29 ± 3 mV, Na-SnSe; −22 ± 5 mV, K-SnSe; −27 ± 4 mV), indicating that the surfaces carry a net negative charge. The characteristic sizes depend on the base used. Particles obtained with the TMA precursors are smaller, with average sizes of 40 ± 20 nm, whereas particles prepared with the alkali precursors show significant size dispersion, with average sizes of approximately 150 ± 50 nm. This difference suggests that TMA^+^ and the alkali cations interact differently with the SnSe surfaces during nucleation and growth. Because TMA^+^ is bulkier (322 pm) and has a more diffuse positive charge than Li^+^, Na^+^, and K^+^, it is expected to exhibit weak, nonspecific interactions with the negatively charged SnSe particle surfaces. By contrast, the smaller inorganic alkali cations can approach the SnSe surface more closely and may therefore contribute more effectively to interfacial charge compensation during nucleation and growth, consistent with the larger average particle size observed.

### Structural Evolution During Thermal Processing

Following synthesis and purification, the SnSe powders were annealed to remove any solvent or labile species and consolidated into dense pellets by spark-plasma sintering (SPS). All pellets have relative densities above 90% (Table S1). To examine how the retained cations influence the structural evolution of the material during thermal processing, we performed laboratory X-ray diffraction (XRD) and synchrotron XRD (Figure 1A) measurements of the consolidated materials. The XRD patterns show that all samples correspond to the orthorhombic SnSe structure with *Pnma* space group and do not exhibit additional reflections of crystalline secondary phases (Figures S3–S5 and Tables S2–S4). Additionally, strain was observed in all samples and was found to vary with the alkali species, indicating differences in the defect structure and microstructural features introduced during SPS processing (Figure S6). Concurrently, the lattice parameters vary systematically with alkali ion identity and with anisotropic variations (Figure 1B,C), indicating that Li^+^, Na^+^, and K^+^ are accommodated differently with the SnSe matrix while preserving the same overall crystal structure. To understand the observed XRD patterns, density functional theory (DFT) calculations were carried out to evaluate the possible alkali incorporation pathways. Since SnSe intrinsically contains Sn vacancies, providing accessible sites within the lattice, substitutional, interstitial, and intercalation configurations was considered. The substitutional model best matches the experimental trends, indicating that vacancy-assisted substitution is the dominant incorporation mechanism. In the case of Li^+^, however, additional interstitial incorporation remains plausible because of its small ionic size (Figure S7).

**Figure 1:**
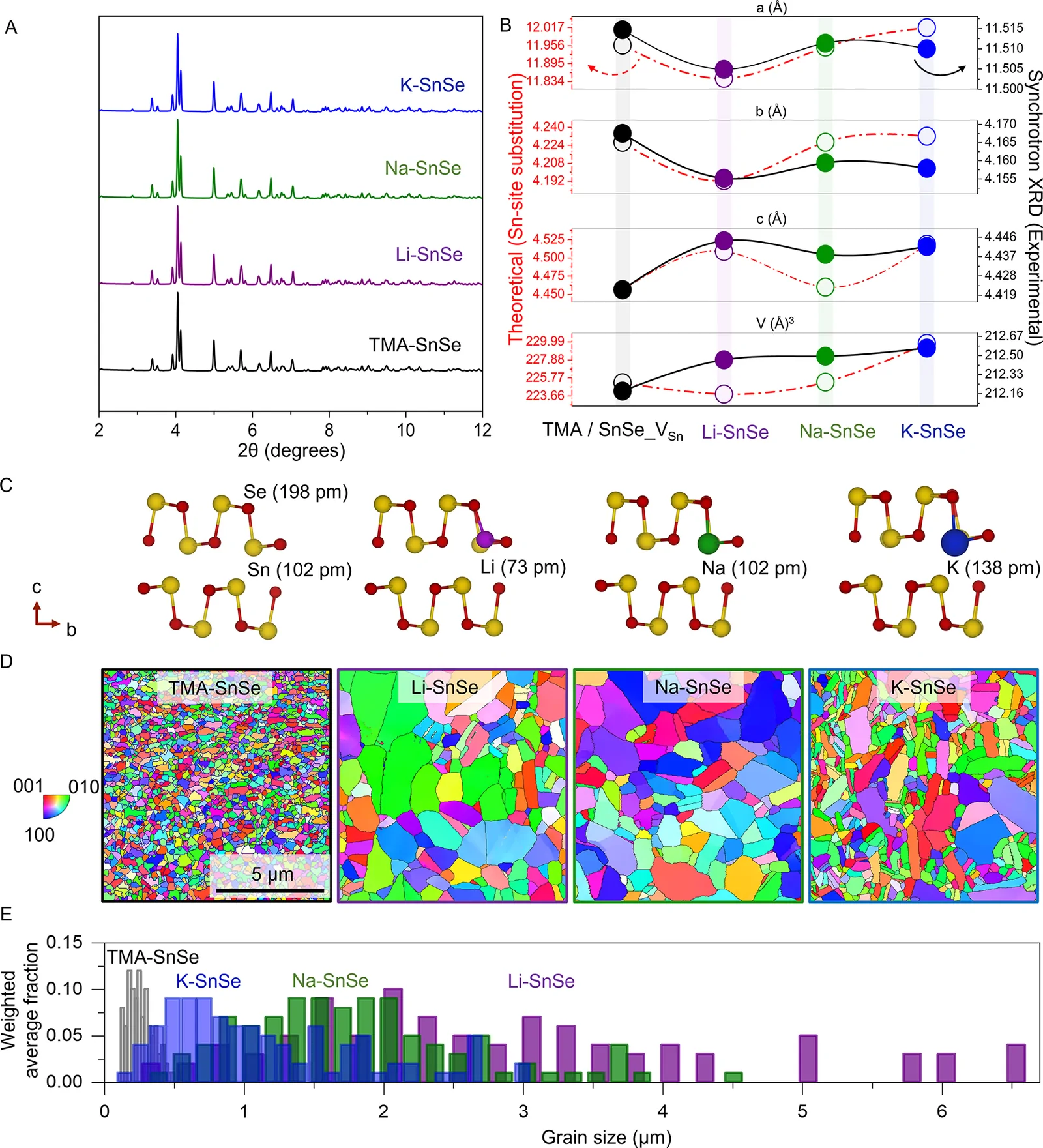
Structural characterization of the TMA-SnSe, Li-SnSe, Na-SnSe, and K-SnSe pellets: (A) synchrotron XRD spectrum of the ground pellets with the X-ray wavelength of 0.207377 Å at room temperature; (B) experimental lattice parameters, calculated from synchrotron XRD patterns and DFT calculations on a 2 × 2 × 2 SnSe supercell; (C) relaxed crystal structure from the DFT calculations of SnSe with different alkali dopants and their corresponding ionic size;^49^ (D) EBSD micrographs of the pellets; (E) weighted average grain size distribution of all pellets.

Whereas the crystal structure remains similar across the series, the microstructure evolves very differently depending on the alkali cation present during synthesis and its redistribution during thermal processing (Figure 1D,E). TMA-SnSe exhibits a fine and comparatively homogeneous grain morphology, whereas all alkali-containing samples show substantial grain coarsening. Li-SnSe displays the most pronounced grain size heterogeneity, with isolated very large grains embedded in a finer matrix, consistent with an incipient abnormal grain growth regime.^48^ In Na-SnSe and K-SnSe, grain coarsening is also pronounced, but the microstructures are better described as heterogeneous grain growth than as clear abnormal grain growth. These observations indicate that the retained alkali ions do not enhance grain growth uniformly but instead generate spatial differences in grain boundary mobility that allow only a subset of grains to grow disproportionately during annealing and SPS.

To understand the origin of these differences in grain growth, we examined the consolidated materials by transmission electron microscopy (TEM) together with local compositional analysis. Because Li cannot be identified by conventional energy-dispersive X-ray spectroscopy (EDX), atom probe tomography was used for Li-SnSe, whereas EDX mapping was used for TMA-SnSe, Na-SnSe, and K-SnSe. TMA-SnSe provides the reference microstructure, showing comparatively thin grain boundaries and absence of the chemically distinct boundary regions observed in the alkali-containing samples (Figures 2A, and S8). By contrast, Li-SnSe, Na-SnSe, and K-SnSe all exhibit microstructures containing dislocations, dislocation pile-ups, and line defects, together with alkali-rich segregation at the grain boundaries and nanoscale regions (Figures 2B–D, and S9 and 10). The extent of alkali segregation at grain boundaries differs strongly across the series. In Li-SnSe and Na-SnSe, the chemically distinct grain boundary regions are typically about 5–12 nm thick, whereas K-SnSe develops much thicker alkali-rich boundary regions of about 20–85 nm. Likewise, the nanoscale alkali-containing domains are finest in Li-SnSe, ranging from 2–7 nm, to somewhat larger in Na-SnSe, 2–11 nm, and coarser in K-SnSe, 6–14 nm.

**Figure 2:**
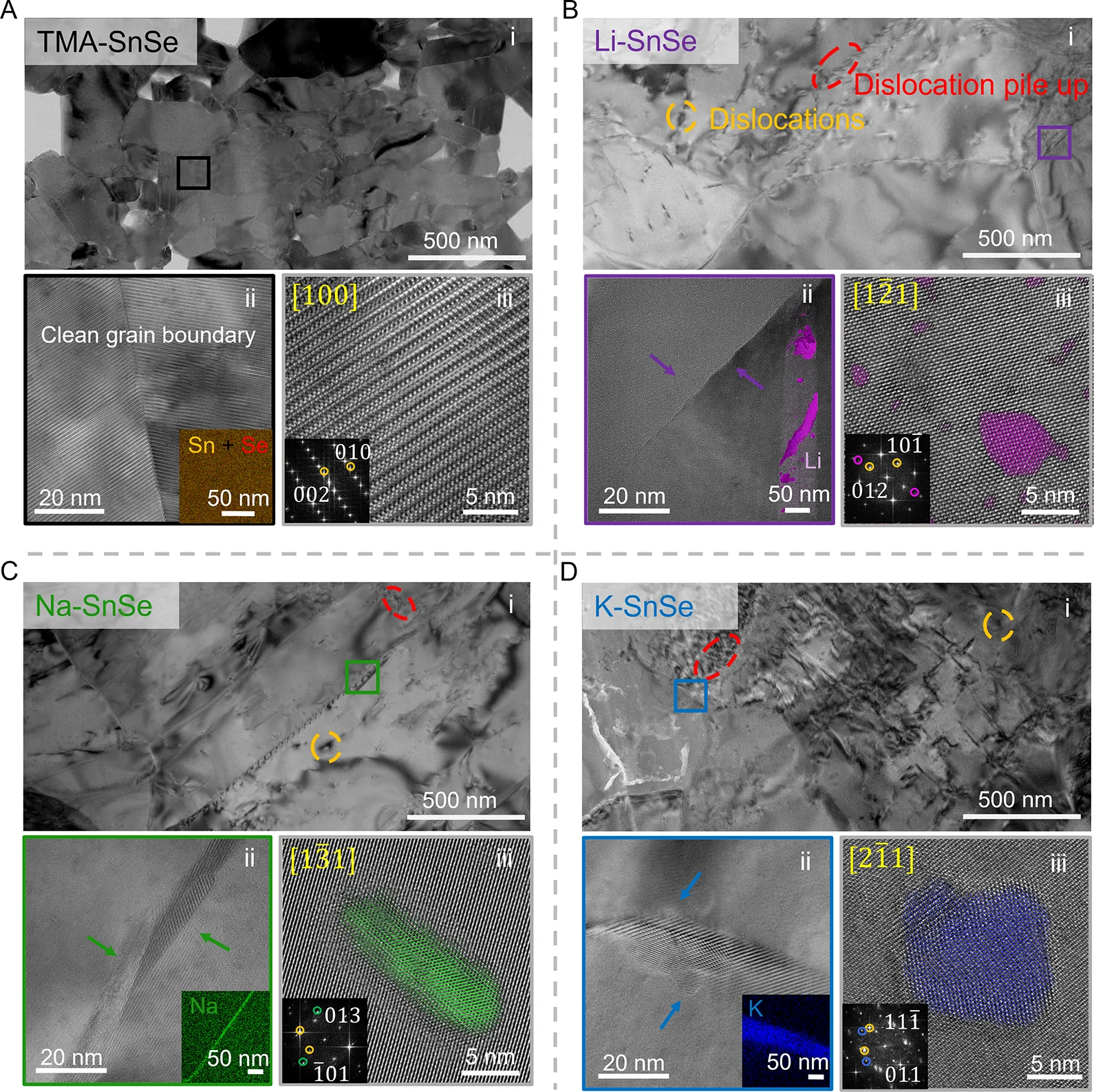
Nanostructural characterization of (A) TMA-SnSe, (B) Li-SnSe, (C) Na-SnSe, and (D) K-SnSe where (i) TEM micrographs show the types of defects present where dislocations are highlighted with orange circles and dislocation pile-ups with red circles. (ii) TEM with EDX micrographs in the inset, showing the elemental distribution of Sn, Se, Na, and K at the grain boundary, and for Li, B­(ii) shows the APT of Li instead. (iii) High-resolution TEM micrographs of the nanoprecipitates with the IFFT images overlaid. The IFFT images were obtained from the FFT spots (inset) from the corresponding-colored circles.

These results show that the decisive variable is not the presence of alkali ions in the matrix, at grain boundaries, or in segregated nanoscale regions (Figure S11) since all three alkali-containing samples exhibit these same classes of environments. Instead, the final microstructure appears to be governed by how each cation partitions among available defects during annealing and SPS processing and how this partitioning modifies grain boundary migration. This can be rationalized using Hume–Rothery considerations, whereby limited solid solubility at substitutional sites promotes the occupation of alternative defects such as vacancies or grain boundaries, leading to distinct microstructural evolution.^50^ We therefore propose that Li leads to the most heterogeneous spatial distribution within the evolving SnSe matrix, resulting in pronounced compositional contrast at grain boundaries and the formation of nanoscale Li-enriched regions. This behavior is consistent with its higher mobility and ability to access multiple competing crystallographic environments, including substitutional, interstitial, and intercalation-type sites, with no single environment being strongly preferred. Because the outcome is therefore sensitive to local microstructural conditions, such as local defect density and degree of supersaturation, different regions of the material can favor different partitioning pathways, lattice incorporation, grain boundary segregation, or nanoscale precipitation during annealing and SPS processing, producing a spatially heterogeneous elemental distribution rather than a uniform one. Such heterogeneity is expected to generate large contrasts in grain boundary mobility, allowing only a subset of grains to grow disproportionately and thereby producing the broad grain size distribution^48,51^ observed in Li-SnSe.

K, by contrast, appears to be less compatible with the SnSe matrix, consistent with its larger ionic size, which leads to increased lattice mismatch and a stronger thermodynamic driving force for interfacial segregation, resulting in thicker chemically distinct grain boundary regions. Electronegativity decreases across the alkali series from Li to Na to K, in parallel with increasing ionic size, so K is both the largest and least electronegative of the three cations. In grain boundary segregation theory, both atomic size misfit and electronic/chemical factors such as electronegativity are recognized as contributing to a solute’s segregation tendency.^52,53^ While this framework has been established primarily for metallic systems, we suggest that an analogous picture may apply here: the combination of large size and low electronegativity for K^+^ would be expected to further disfavor substitutional incorporation and favor segregation to grain boundaries, consistent with the pronounced K-rich interfacial regions observed experimentally. This behavior is consistent with segregation-dominated grain boundary modification,^54,55^ where solute drag rather than particle pinning governs boundary migration.^56,57^ The resulting reduced boundary mobility limits grain growth while giving rise to thick alkali-rich interfacial regions. Na lies between these two limits, showing a more balanced partitioning between matrix incorporation, grain boundary segregation, and nanoscale precipitation and consequently a substantial but less extreme grain coarsening. Across this series, ionic size appears to be the dominant factor governing lattice strain, defect accommodation, and site accessibility, while differences in electronegativity provide a secondary, reinforcing contribution to alkali–SnSe bonding.

All together, these results indicate that alkali ions do not act simply as conventional bulk dopants. Rather, their identity controls how retained cations redistribute during processing, and this redistribution defines the defect structure and grain growth behavior of the final SnSe material.

### Transport Properties

Having established how alkali ions influence the microstructure of polycrystalline SnSe, we examined how these structural changes affect thermoelectric properties, including the Seebeck coefficient (*S*), electrical conductivity (σ), and thermal conductivity (κ_total_ = κ_lattice_ + κ_electronic_). Because SnSe is structurally anisotropic and SPS introduces texture, transport was measured in parallel and perpendicular to the pressing axis. The measurements were found to be highly reproducible across independently prepared batches, confirming the robustness of the observed transport behavior (Figure S12). As the parallel direction exhibits the highest performance (Figure S13), the discussion below focuses on this orientation, with the perpendicular data provided in the Supporting Information (Figure S14).

At 300 K, σ follows the trend Li-SnSe > Na-SnSe > K-SnSe > TMA-SnSe (Figure 3A), whereas the carrier concentration (p_*H*_) follows Na-SnSe: 2.3 × 10^19^ cm^–3^, K-SnSe: 8.1 × 10^18^ cm^–3^, Li-SnSe: 5.13 × 10^18^ cm^–3^, TMA-SnSe: 2.8 × 10^16^ cm^–3^. The much lower σ of TMA-SnSe is consistent with its far lower carrier concentration. By contrast, Li-SnSe shows the highest σ among the alkali-containing samples, despite its lower p_*H*_ than Na-SnSe, indicating that differences in carrier mobility dominate over carrier concentration. This interpretation is supported by its higher weighted mobility (μ_w_, Figure 3B) and lower potential barrier (*E*_*b*_: σ ∝ T^–1/2^ exp (-*E*_*b*_/*k*_B_ *T*)). For Li-SnSe, this barrier is ca. 54 meV, whereas it is ca. 184 meV for Na-SnSe and ca. 192 meV for K-SnSe.

**Figure 3:**
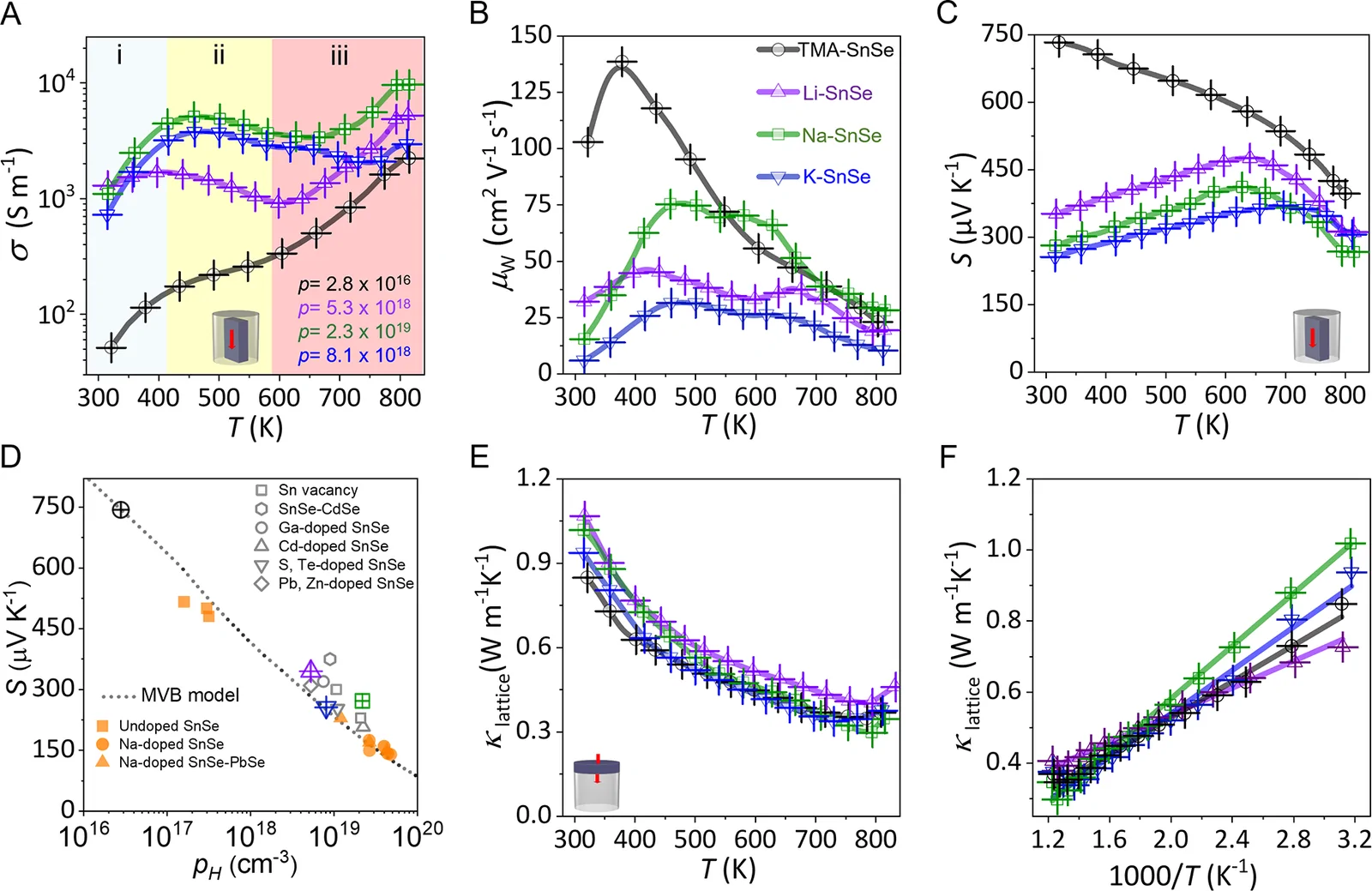
Transport properties of the parallel direction to the pressing axis for pellets prepared with the different alkali ions: (A) electrical conductivity (σ), with three distinct temperature-dependent transport regimes (i–iii) indicated and the carrier concentration (*p*, cm^–3^) shown in the bottom-right part, (B) weighted mobility (μ_*w*_), (C) Seebeck coefficient (S), (D) Pisarenko plot at 300 K, including solid-state references (orange dots),^40,61,65–69^ solution-processed references (black dots),^15,18,70–74^ and a dashed line (calculated using a multiband model (MVB))^75^ reproduced from ref 16. Available under CC-BY 4.0. Copyright 2024, Liu, Y.; Lee, S. et al. (E) Lattice thermal conductivity (κ_lattice_) and (F) κ_lattice_ as a function of 1000/T.

This functional form describes thermionic emission-type transport over a potential barrier without specifying its physical origin. While the pronounced barrier in K-SnSe is consistent with its markedly wider grain boundary regions, nanoscale precipitate size follows the same increasing trend as Eb across the series (2–7, 2–11, and 6–14 nm for Li-, Na-, and K-SnSe, respectively), suggesting that alkali-enriched precipitates contribute to the extracted barrier alongside grain boundaries. This is further supported by the comparable barrier heights obtained from transport measurements performed perpendicular to the SPS-pressing direction where Li-SnSe is ca. 54 meV, whereas it is ca. 185 meV for Na-SnSe and *ca*. 191 meV for K-SnSe.

All samples exhibit a positive *S* over the full temperature range, indicating p-type transport (Figure 3C). TMA-SnSe, which has the lowest p_*H*_, shows the highest *S* at low temperatures, whereas the alkali-containing samples show lower values consistent with their higher p_*H*_. At 300 K, *S* of the alkali-containing samples lies above the multiband Pisarenko relationship (Figure 3D). This is consistent with an additional contribution from boundary-related carrier filtering or other microstructural effects.^31,58–60^ K-SnSe, however, departs from this trend. Despite a carrier concentration of the same order of magnitude as Li-SnSe and Na-SnSe, its *S* falls essentially on the Pisarenko baseline, showing no resolvable enhancement. The much thicker K-rich regions behave as extended diffusive scattering layers, degrading mobility across the carrier energy spectrum without selective filtering of low-energy carriers needed to enhance *S*.

The temperature dependence of σ and *S* shows that all alkali-containing samples pass through the same three transport regimes, although the temperature range over which each regime dominates depends on the alkali ion. At low temperatures, the increase in σ with temperature is consistent with thermally activated transport across potential barriers (Figure 3A­(i)).^16,61,62^ Once these barriers become less limiting, σ decreases with temperature as carrier phonon scattering becomes increasingly important (Figure 3A­(ii)). This crossover occurs at a lower temperature for Li-SnSe than for Na-SnSe and K-SnSe, which is consistent with Li-SnSe already having the weakest potential barriers near room temperature. At even higher temperatures (>600 K), σ turns upward again as intrinsic excitation and bipolar transport become increasingly significant on approaching the *Pnma* to *Cmcm* phase transition (Figure 3A­(iii)). The onset of this upturn appears earlier in Li-SnSe and Na-SnSe, and later in K-SnSe, indicating that the K-rich boundary regions continue to hinder carrier transport at higher temperatures. *S* evolves concurrently. In Li-SnSe, Na-SnSe, and K-SnSe, *S* increases with temperature, reaches a maximum, and then decreases at higher temperatures. The temperature of this maximum broadly tracks with the onset of the σ upturn, indicating that both responses are governed by the same crossover from majority carrier transport to increasingly bipolar transport. Overall, the electronic transport data show that Na gives the best balance between carrier concentration and mobility, Li gives the highest room-temperature mobility because it is least hindered by interfacial barriers, and K experiences the strongest mobility penalty due to segregation at chemically broad grain boundary regions.

By contrast, the differences in thermal transport are comparatively modest (Figure 3E). All samples retain low κ_total_ over the full temperature range, as expected for the strong lattice anharmonicity in SnSe. After subtraction of the electronic contribution, κ_lattice_ shows an approximately linear dependence on 1/T over most of the measured range, consistent with dominant Umklapp scattering (Figure 3F). An additional contribution from bipolar transport emerges at higher temperatures due to thermally activated carriers.^16,61,62^ However, despite the clear microstructural differences, the κ_lattice_ values remain relatively similar across the series, indicating that the phonons that dominate heat transport are not affected as strongly as charge carriers by the alkali-dependent changes in microstructure.^63,64^

In summary, the thermoelectric performance is therefore determined primarily by how effectively each alkali increases the hole concentration without introducing a severe mobility penalty at the interfaces. Na-SnSe provides the best balance. It raises p_H_ most effectively while avoiding the strong boundary-limited transport seen in K-SnSe and therefore gives the highest overall performance (Figure S13). Li-SnSe benefits from the highest room-temperature mobility and the weakest apparent grain boundary barriers, but its lower carrier concentration limits the overall gain (Figure S15). K-SnSe, by contrast, is most strongly penalized by broad K-rich grain boundary regions, which reduce mobility without a compensating enhancement in *S*. These results reinforce the structural analysis: alkali ions do not influence transport simply through conventional bulk doping but through the way their identity controls partitioning between matrix, grain boundaries, and segregated nanoscale regions during processing.

## Conclusion

In this work, we show that alkali ions introduced during solution synthesis remain active throughout the conversion of SnSe powders into dense polycrystalline materials, where they direct the evolution of the grain morphology, defect distribution, and transport properties. Li^+^, Na^+^, and K are found in the matrix, at grain boundaries, and in segregated nanoscale regions, but their distributions vary with cation identity, in both relative proportions and characteristic length scales. Li yields the strongest grain size heterogeneity and the weakest apparent grain boundary barriers, consistent with a redistribution pathway that promotes large local contrasts in the grain boundary mobility. Na provides a more balanced partitioning between matrix incorporation, grain boundary segregation, and nanoscale precipitation and consequently provides the best overall combination of carrier concentration, mobility, and thermoelectric performance. K, by contrast, results in the thickest chemically distinct grain boundary regions and the strongest interfacial penalty to carrier transport, consistent with a greater tendency toward segregation and solute drag.

More broadly, these results show that alkali ions in solution-processed SnSe cannot be understood simply as conventional bulk dopants or as transient synthetic species. Their impact is defined by how they are retained from synthesis and how they are redistributed during annealing and densification. In this sense, the final thermoelectric properties are governed not only by carrier concentration but also by the full microstructural legacy of the precursor chemistry. This insight is relevant beyond SnSe itself as many functional materials are prepared from solution-synthesized particles that undergo substantial structural and chemical reorganization during later processing. Establishing reliable structure–property relationships in such systems, therefore, requires attention not only to the final composition but also to the pathway by which residual species partition between matrix, boundaries, and segregated domains during material evolution.

## Supplementary Material



## Notes

SEM micrographs, XRD, Rietveld refinement of synchrotron XRD, DFT calculations, STEM-HAADF micrographs, STEM-EDX micrographs, APT analysis, thermoelectric transport measurements, additional transport calculations, and relative densities of all samples (PDF)
