# Supplementary material for: Alkali Ions Retained from Synthesis Govern Microstructure and Transport in Solution-Processed SnSe
Source: Chem Mater. 2026 Sep 8;38(18):9323–32. doi: 10.1021/acs.chemmater.6c01284 (PMC13603064; doi:10.1021/acs.chemmater.6c01284)
Supplement: Supplementary file 1 [file cm6c01284_si_001.pdf]

# Supporting Information

# Alkali Ions Retained from Synthesis Govern Microstructure and Transport in Solution-Processed SnSe

Christine Fiedler, [a] Sharona Horta, [a] Navita Jakhar, [a] Krishnendu Maji, [a] Tobias Kleinhanns, [a] Jordi Llorca, [b] Qi Wang, [a] Maria Garcia-Ramon, [a] Sambit Das, [c] Vikram Gavini, [c] Yu Liu, [a],[d]* Maria Ibáñez [a]*

[a] Institute of Science and Technology Austria, Am Campus 1, 3400 Klosterneuburg, Austria

[b] Department of Chemical Engineering and Barcelona Research Center in Multiscale Science and Engineering, Universitat Politècnica de Catalunya, EEBE, Av. Eduard Maristany 10-14, Barcelona 08019, Catalonia, Spain

[c] University of Michigan, 503 Thompson Street, Ann Arbor, MI 48104, United States

[d] Anhui Province Engineering Research Center of Flexible and Intelligent Materials, School of Chemistry and Chemical Engineering, Hefei University of Technology, 230009 Hefei, China

*Email: mibanez@ista.ac.at.

*Email: yliu@hfut.edu.cn.

# CONTENTS

**Chemicals** ........................................3
**Pisarenko relation**........................................3
**References** ........................................23

## Chemicals

Tin (II) chloride dihydrate ($SnCl_2 \cdot 2H_2O$, 98%), sodium hydroxide (NaOH, pellets 98%) and sodium borohydride ($NaBH_4$, 98%), were purchased from Fisher Scientific. Selenium powder (Se, 100 mesh, and ≥99.5%), lithium hydroxide (LiOH, ≥ 98%), lithium borohydride ($LiBH_4$, ≥90%), potassium hydroxide (KOH, 99.99%), tetramethylammonium hydroxide pentahydrate (TMAOH, ≥95%) and ethanol (95%) were purchased from Sigma-Aldrich. Potassium borohydride ($KBH_4$, >98%) was purchased from Thermo Fisher Scientific. Tetramethylammonium borohydride ($TMABH_4$, >90%) was purchased from Tokyo Chemical Industry (TCI). Milli Q-Water (Millipore) was used in the SnSe NP synthesis and purification. All chemicals were used as received without further purification. Syntheses were carried out using a standard Schlenk line.

## Pisarenko relation

The Pisarenko relation and multivalence band (MVB) framework used in this work describe the correlation between the Seebeck coefficient ($S$) and carrier concentration ($n$) in thermoelectric materials. In this approach, the Seebeck coefficient is expressed as $S = (kB/e)[A - \ln(n)]$, where $S$ is the Seebeck coefficient ($V\ K^{-1}$), n is the carrier concentration ($cm^{-3}$), kB is the Boltzmann constant ($1.380649 \times 10^{-23}\ J\ K^{-1}$), e is the elementary charge ($1.602176 \times 10^{-19}$ C), and A is a material-dependent constant that incorporates contributions from scattering processes, band structure effects, and transport parameters.

The Pisarenko plot is constructed by plotting the experimentally determined $S$ as a function of $\log(n)$ or $\ln(n)$, depending on the convention used. In this representation, the slope is related to the universal factor kB/e, while shifts in the intercept reflect changes in the electronic structure and transport characteristics.

The multivalence band (MVB) model further relates transport properties through the concept of weighted mobility ($\mu_w$), where $\sigma = ne\mu$ and $\mu_w = \mu$ $(m^*/m_e)^{3/2}$. In this expression, $\mu$ is the charge carrier mobility, me is the free electron mass ($9.109383 \times 10^{-31}$ kg), and m* represents the effective mass parameter associated with the electronic transport behavior. Within this framework, changes in the Pisarenko intercept are interpreted in terms of variations in weighted mobility, which captures combined effects of carrier mobility and electronic structure.

Experimentally, the Pisarenko plot was obtained by plotting the measured *S* against *n* determined from Hall measurements. The resulting trends were analyzed using linear fitting in log(*n*) space, and deviations from reference behavior were used to assess changes in weighted mobility and electronic transport arising from alkali incorporation.

The constants used are kB = $1.380649 \times 10^{-23}$ J $K^{-1}$, e = $1.602176 \times 10^{-19}$ C, h = $6.626070 \times 10^{-34}$ J s, and $m_e = 9.109383 \times 10^{-31}$ kg.

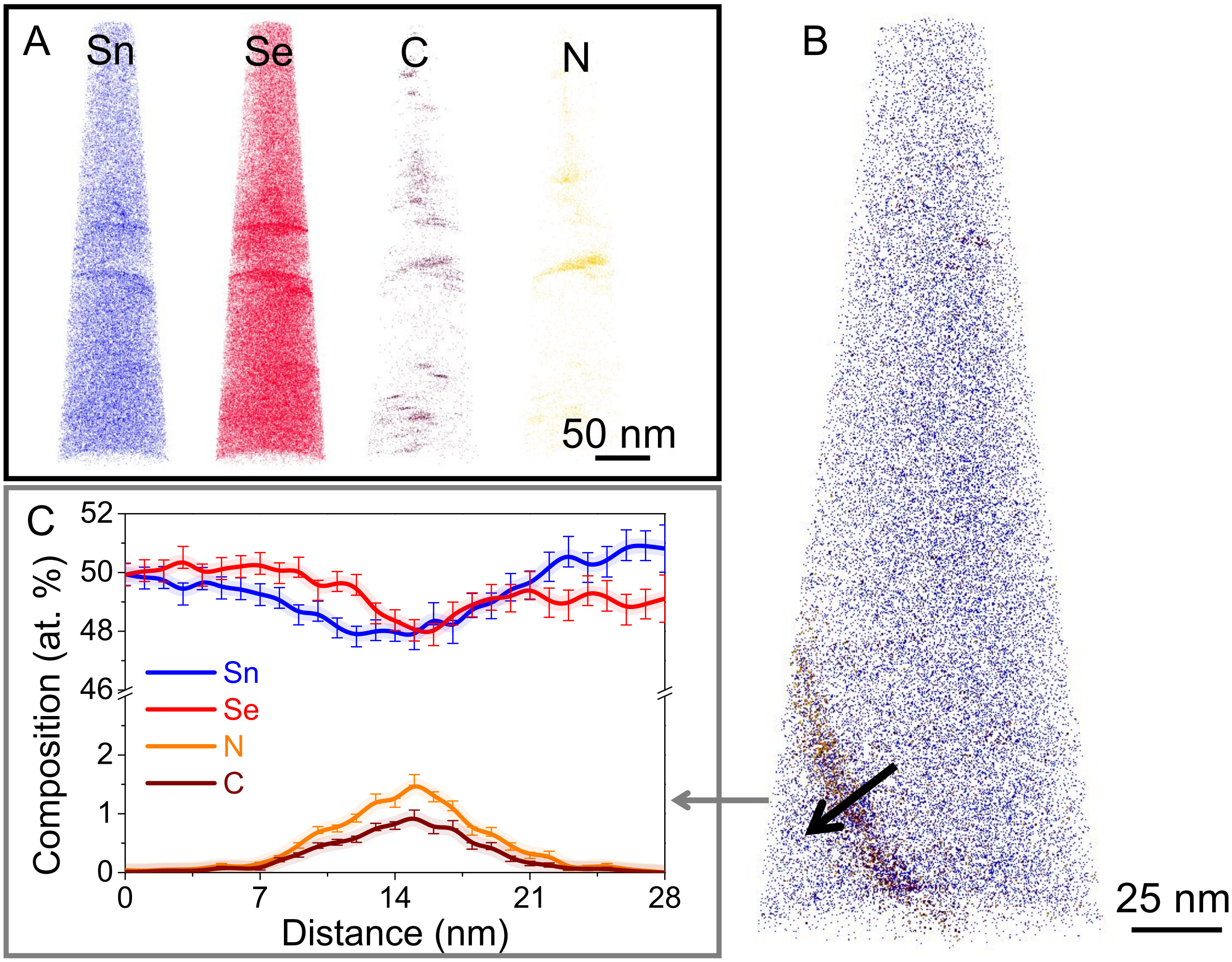


**Figure S1.** APT of a TMA-SnSe pellet where (A) shows the 3D distribution of each element. Sn and Se show an almost homogeneous distribution, and very small traces of N and C in the final pellet present aggregations in the grain boundary. (B) The 1D composition profile and (C) the composition proximity histogram of the grain boundary shows that the maximum enrichment of C and N at this grain boundary is <2%.

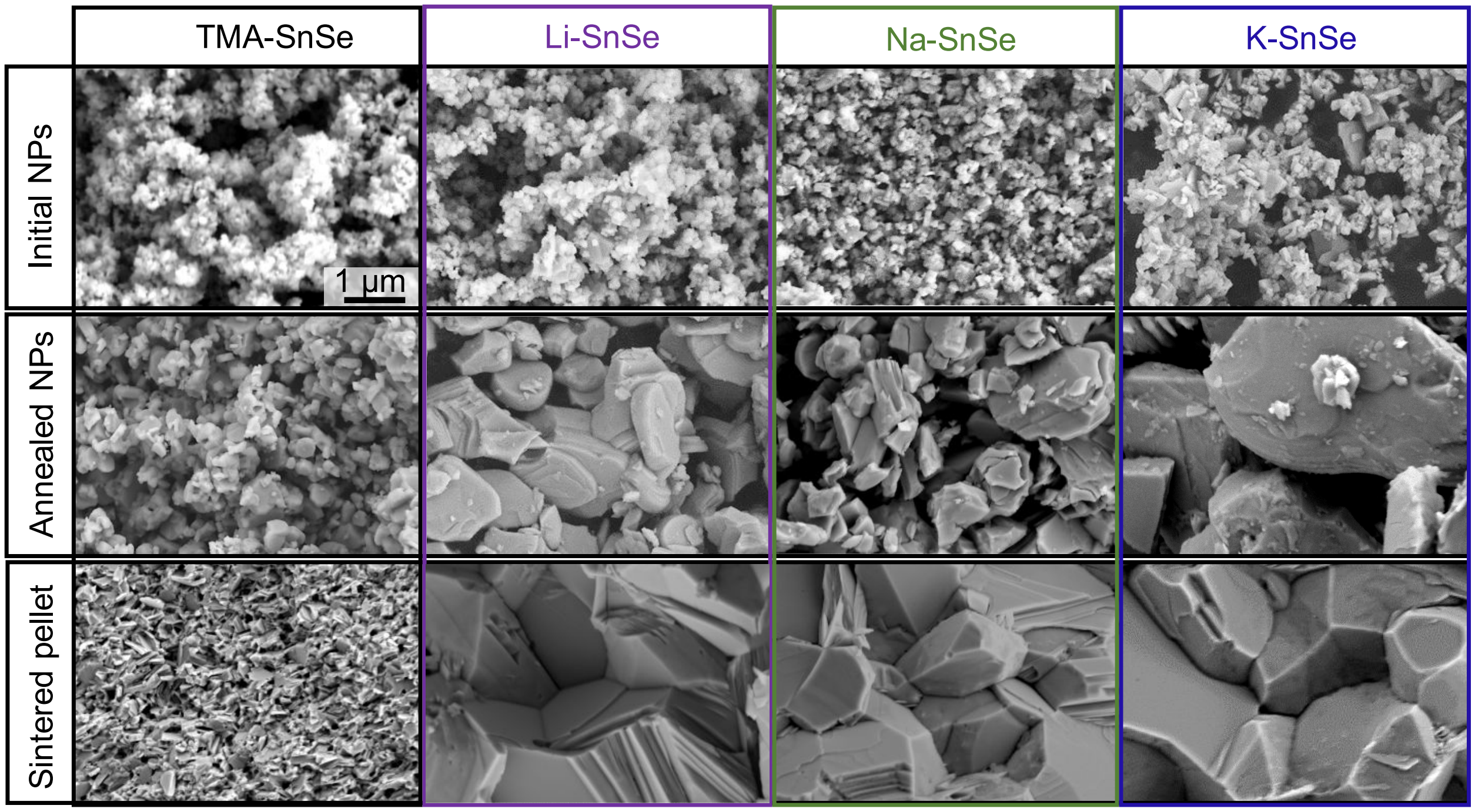


**Figure S2.** SEM images of the powders before thermal treatment, the powders after thermal treatment, and the corresponding densified pellets for samples prepared with different alkali ions, including TMA-SnSe, Li-SnSe, Na-SnSe, and K-SnSe.

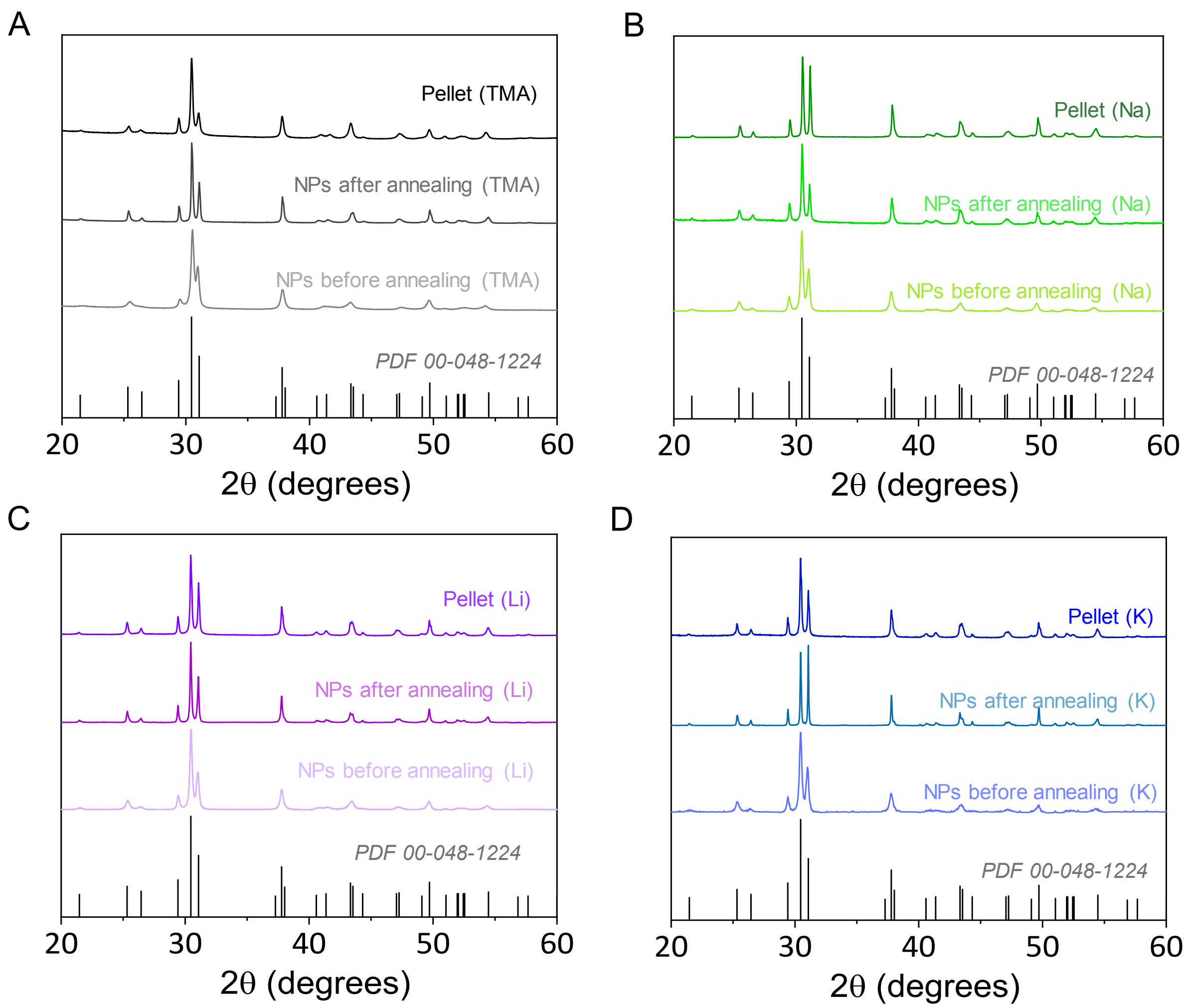


**Figure S3.** XRD patterns of the SnSe particles, annealed powders and consolidated pellets in the in-plane direction made with (A) $TMA^+$ (B) $Na^+$ (C) $Li^+$ and (D) $K^+$.

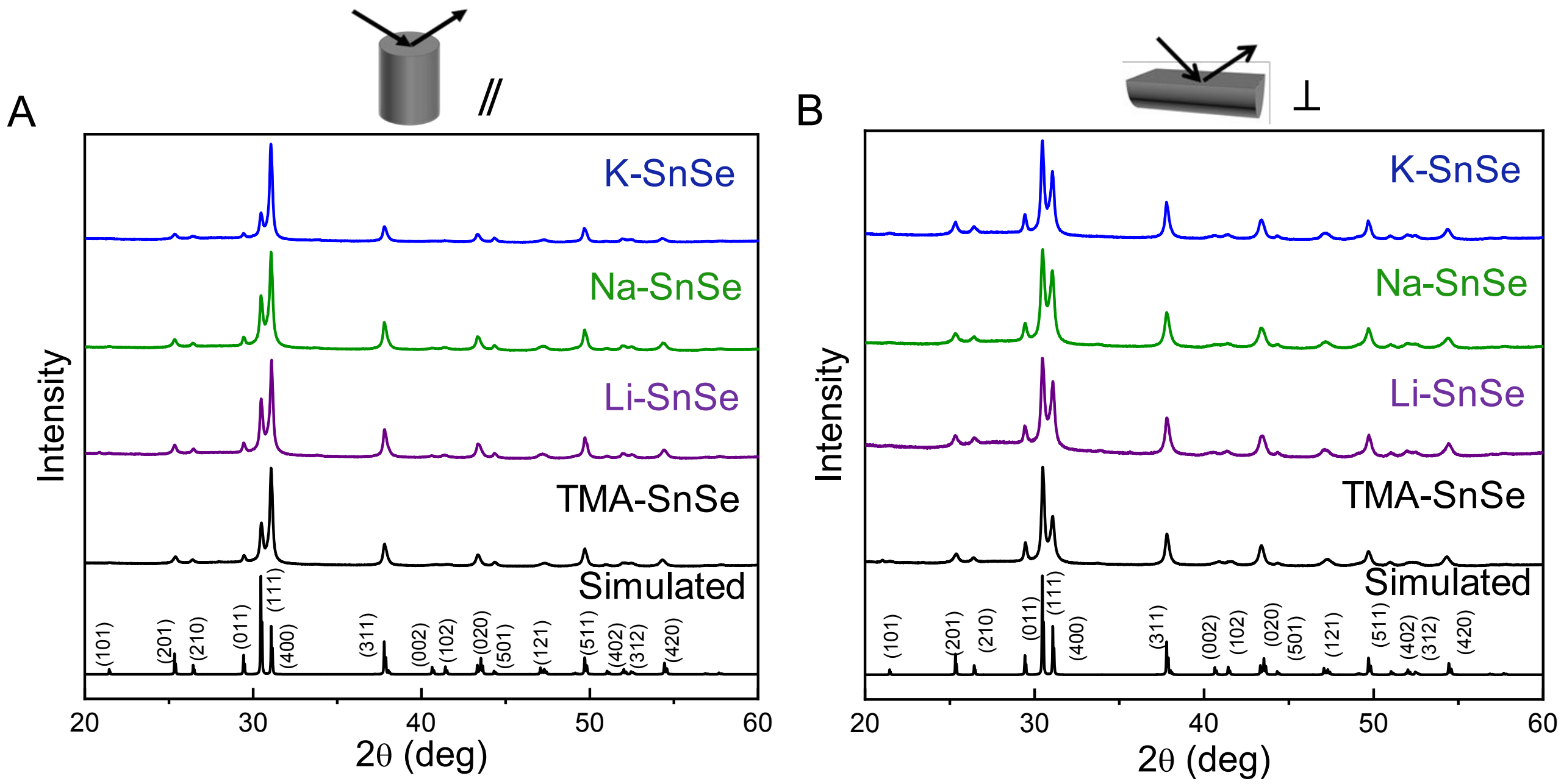


**Figure S4.** Room temperature XRD pattern of all samples where (A) parallel and (B) perpendicular direction to the pressing axis.

The degree of orientation of crystalline grain (texturing) is expressed by the orientation factor from the Lotgering method. From the X-ray diagrams the sum of the intensities of the (h00) reflections and the sum of the intensities of all (hkl) reflections (which occur in a certain range of θ values) are obtained and the ratio of the two sums is calculated for an oriented sample (value p) and for the non-oriented material (value $P_0$).

$$P_0 = \sum I_0(h00)/\sum I_0\,(hkl)$$

$$P = \sum I(h00)/\sum I(hkl)$$

$$F = {}^{P-P_0}\!/_{1-P_0}$$

where, I and $I_0$ are the intensity of the X-ray diffraction peaks of textured and randomly oriented samples, respectively. P and $P_0$ represent the ratios of the sum of intensities of the (h00) reflections to the total intensities of all (hkl) reflections for textured and randomly oriented samples, respectively. the summation is ranging from 20°-60°. To calculate the $P_0$, we have used the standard diffraction pattern of SnSe from Inorganic Crystal Structure Database (Database code: ICSD 12863).

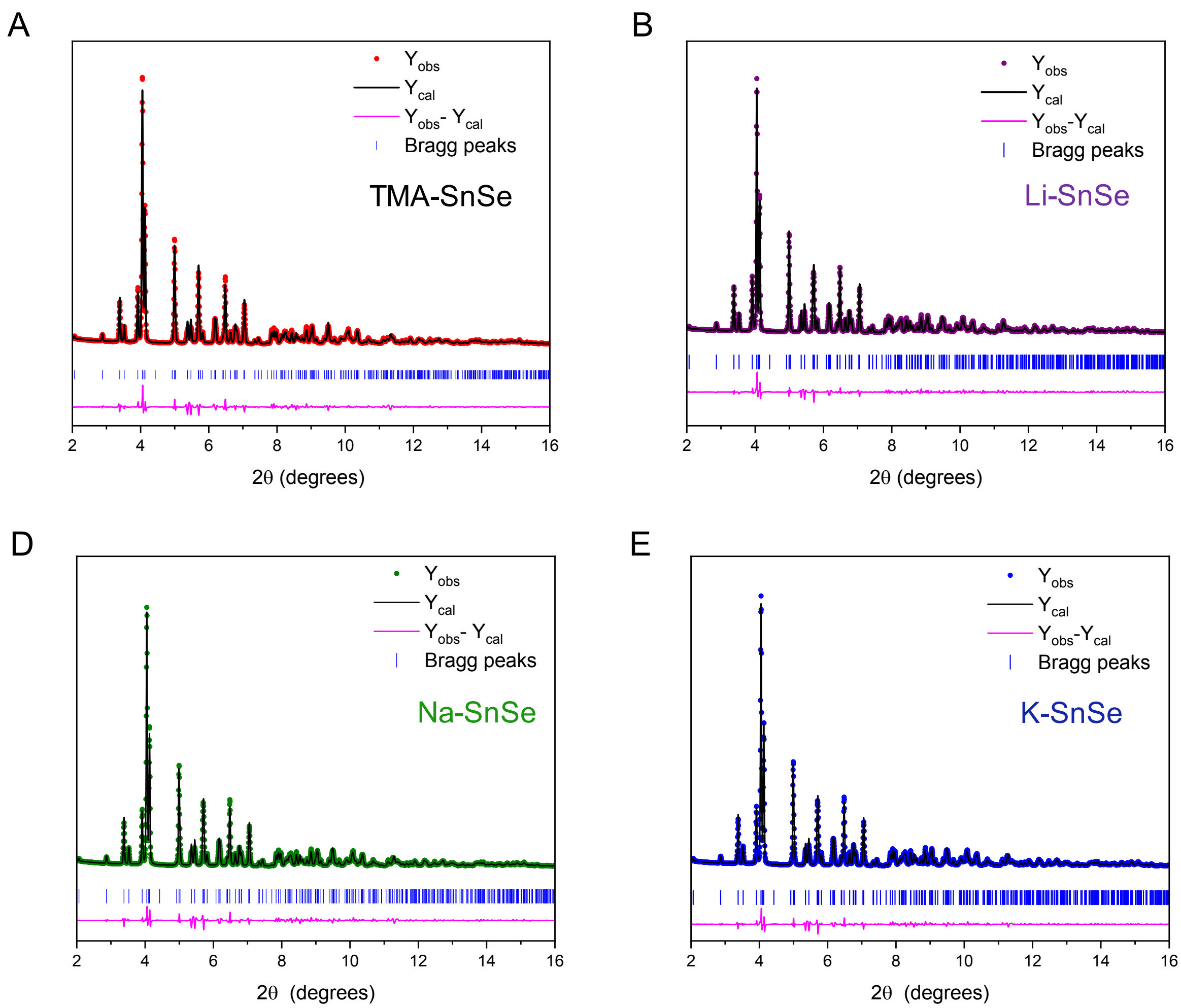


**Figure S5.** Rietveld refinement of synchrotron data of the bulk samples at room temperature (λ= 0.207377 Å) where (A) TMA-SnSe, (B) Li-SnSe, (C) Na-SnSe and (D) K-SnSe.

To quantify the strain present in the samples, Williamson-Hall (W-H) analysis was performed using X-ray diffraction (XRD) data collected both on a laboratory-based diffractometer and synchrotron data.

The broadening of diffraction peaks was analyzed by considering the full width at half maximum (FWHM) of the reflections. Prior to the analysis, instrumental broadening was subtracted from the measured FWHM values to obtain the intrinsic peak broadening arising solely from the samples.

$$\beta \cos(\theta) = \frac{k\lambda}{D} + 4\varepsilon \sin(\theta)$$

where $\beta$ is the corrected FWHM (in radians), $\theta$ is the Bragg diffraction angle, $k$ is the shape factor (taken as 0.9), $\lambda$ is the X-ray wavelength, (D) is the crystallite size, and $\varepsilon$ represents the lattice strain. By plotting $\beta\cos(\theta)$ as a function of $\sin(\theta)$, the above equation can be expressed in the linear form (y = c + mx), where the intercept c corresponds to $\frac{k\lambda}{D}$ and the slope m equals $4\varepsilon$. The lattice strain was subsequently determined from the slope of the linear fit and expressed as a percentage.

The W-H analysis was performed using the peak broadening of the (200), (210), (311), and (420) reflections using synchrotron PXRD data. The estimated crystallite sizes for the TMA-, Li-, Na-, and K-containing samples are 166 nm, 82 nm, 77 nm, and 82 nm, respectively. The corresponding strain values are found to be 0.18%, 0.10%, 0.13%, and 0.12%, respectively. While the strain values are consistent with the expected behavior of the sintered pellets, the crystallite sizes obtained from the synchrotron data differ significantly from those estimated from local microstructural characterization.

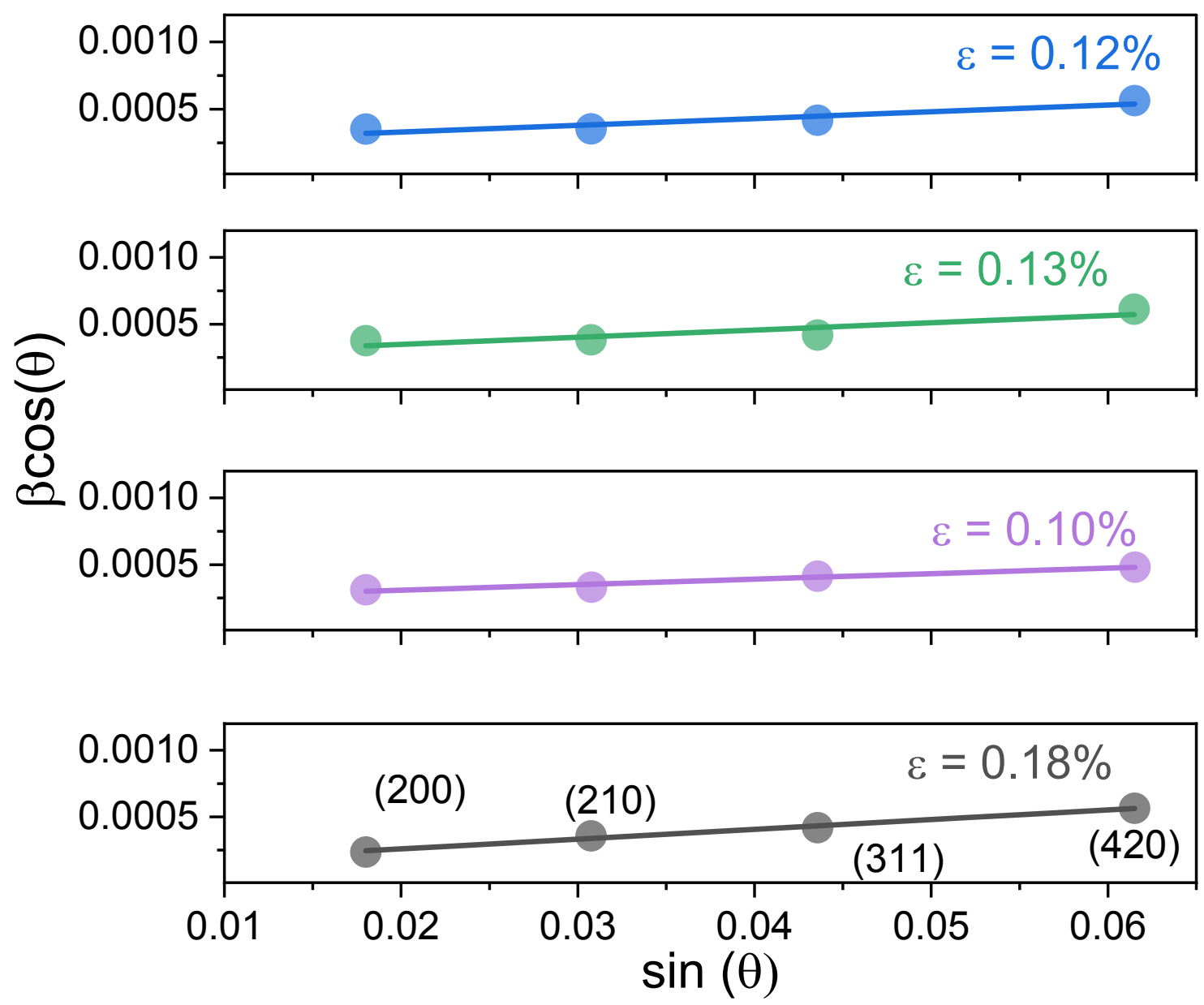


**Figure S6.** Williamson-Hall plot for TMA, Li, Na, and K-doped SPS pellets along with linear fitting and determined micro-strain values for diffraction taken from on synchrotron data.

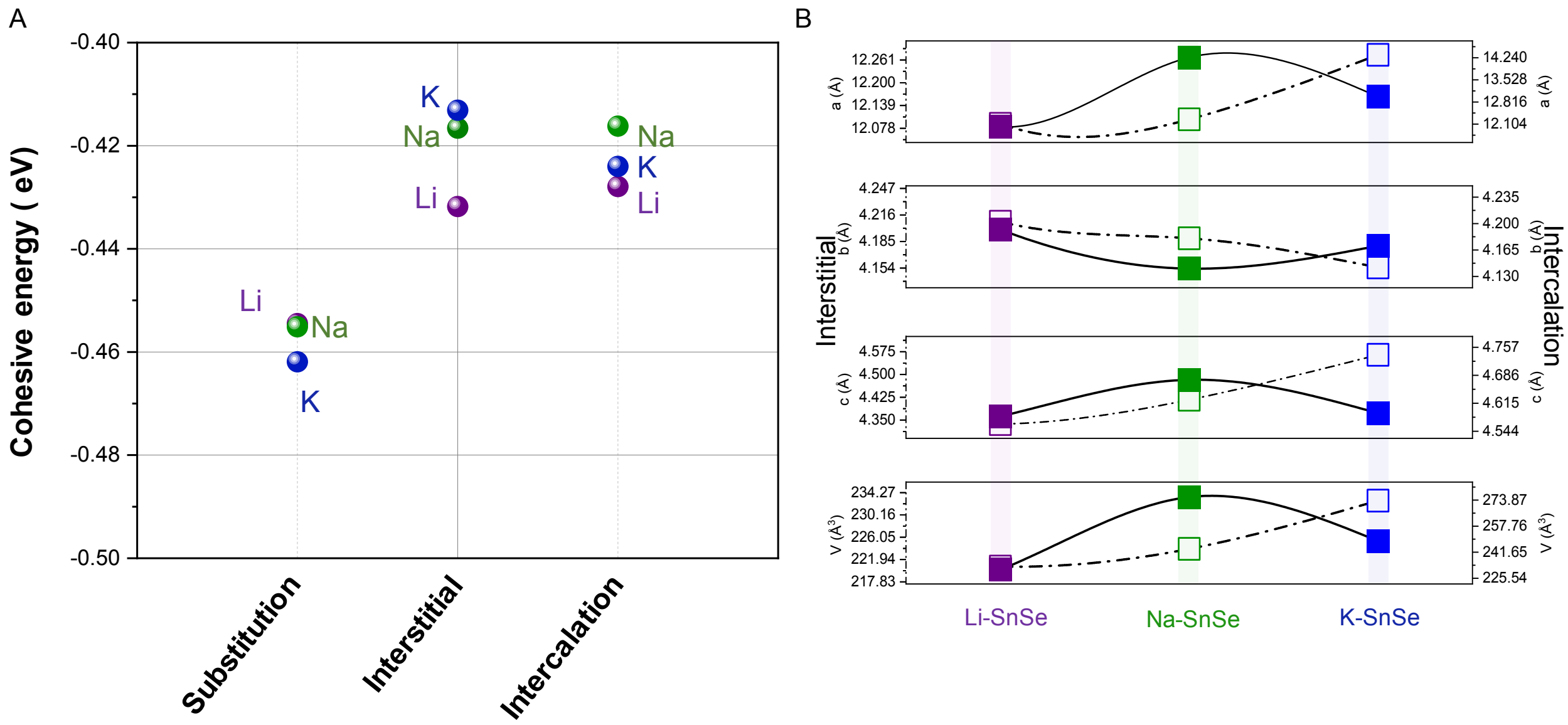


**Figure S7.** DFT calculations for a 2 × 2 × 2 SnSe supercell: (A) cohesive energies for different configurations, and (B) corresponding optimized lattice parameters.

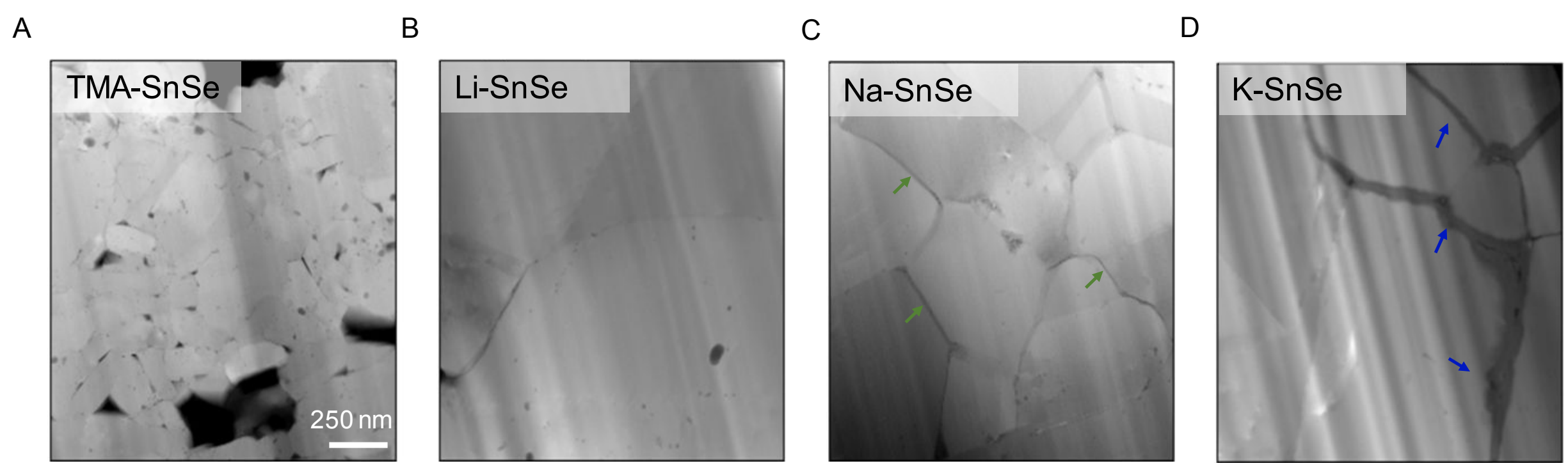


**Figure S8.** STEM-HADDF micrographs of (A) TMA-SnSe, (B) Li-SnSe, (C) Na-SnSe and (D) K-SnSe, where the arrows indicate the thicker grain-boundary regions.

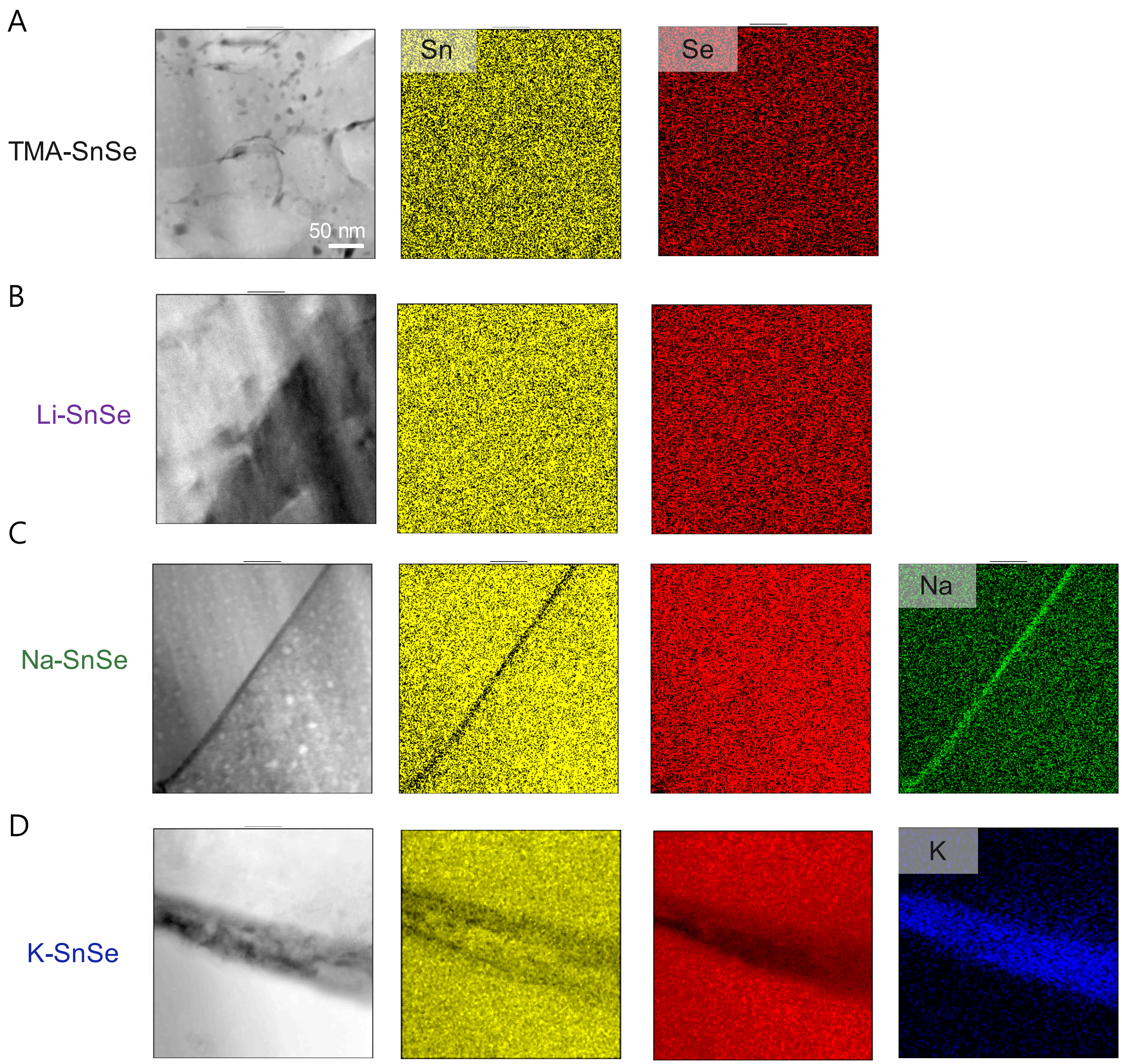


**Figure S9.** STEM-EDX elemental maps of (A) TMA-SnSe, (B) Li-SnSe, (C) Na-SnSe, and (D) K-SnSe. Li is not shown because it cannot be detected by EDX.

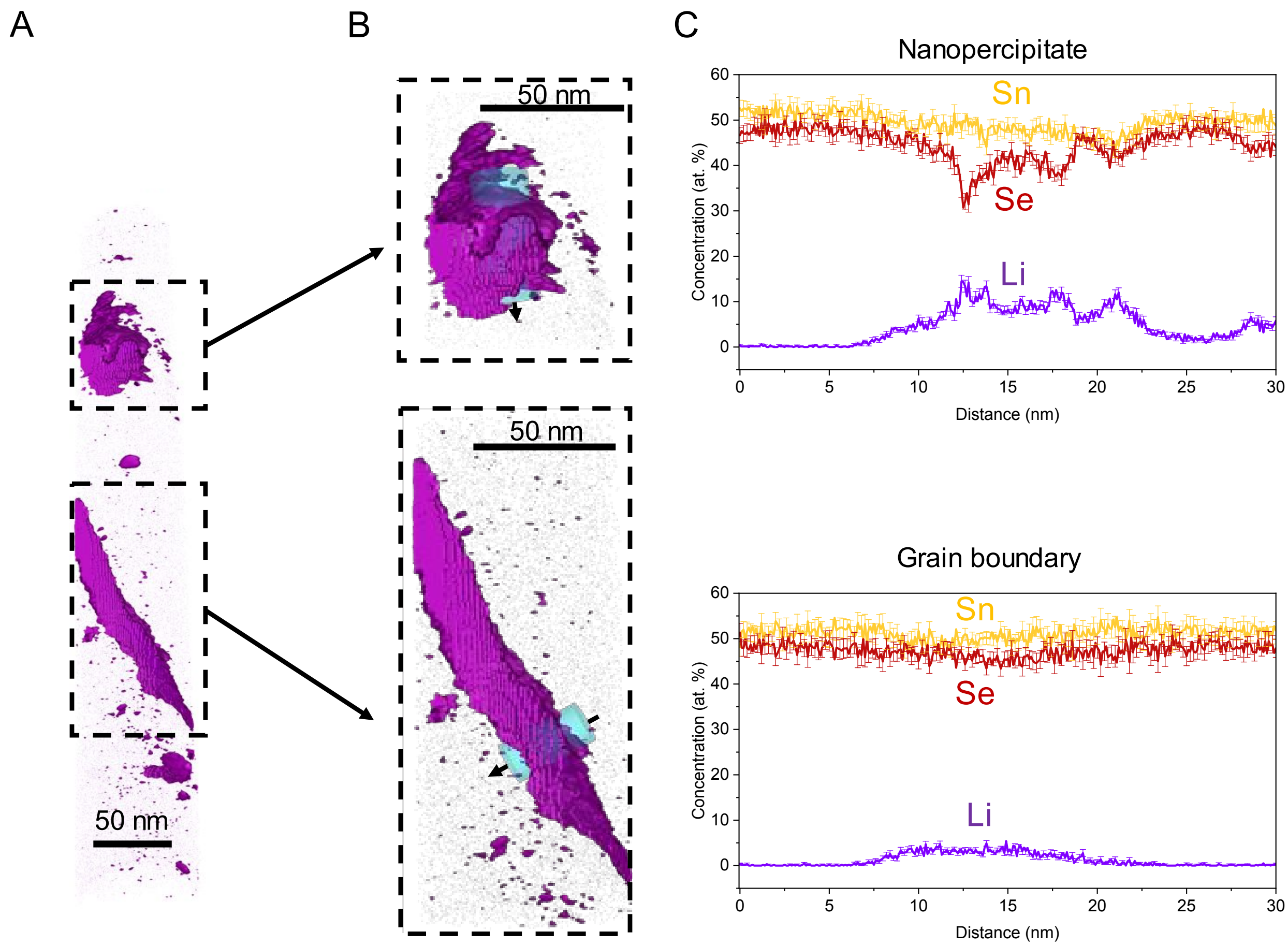


**Figure S10.** APT analysis of Li-SnSe: (A) three-dimensional Li distribution throughout the prepared tip; (B) enlarged views highlighting a Li-rich nanoprecipitate and a Li-enriched grain-boundary region; and (C) concentration profiles of Sn, Se and Li across the nanoprecipitate and grain boundary.

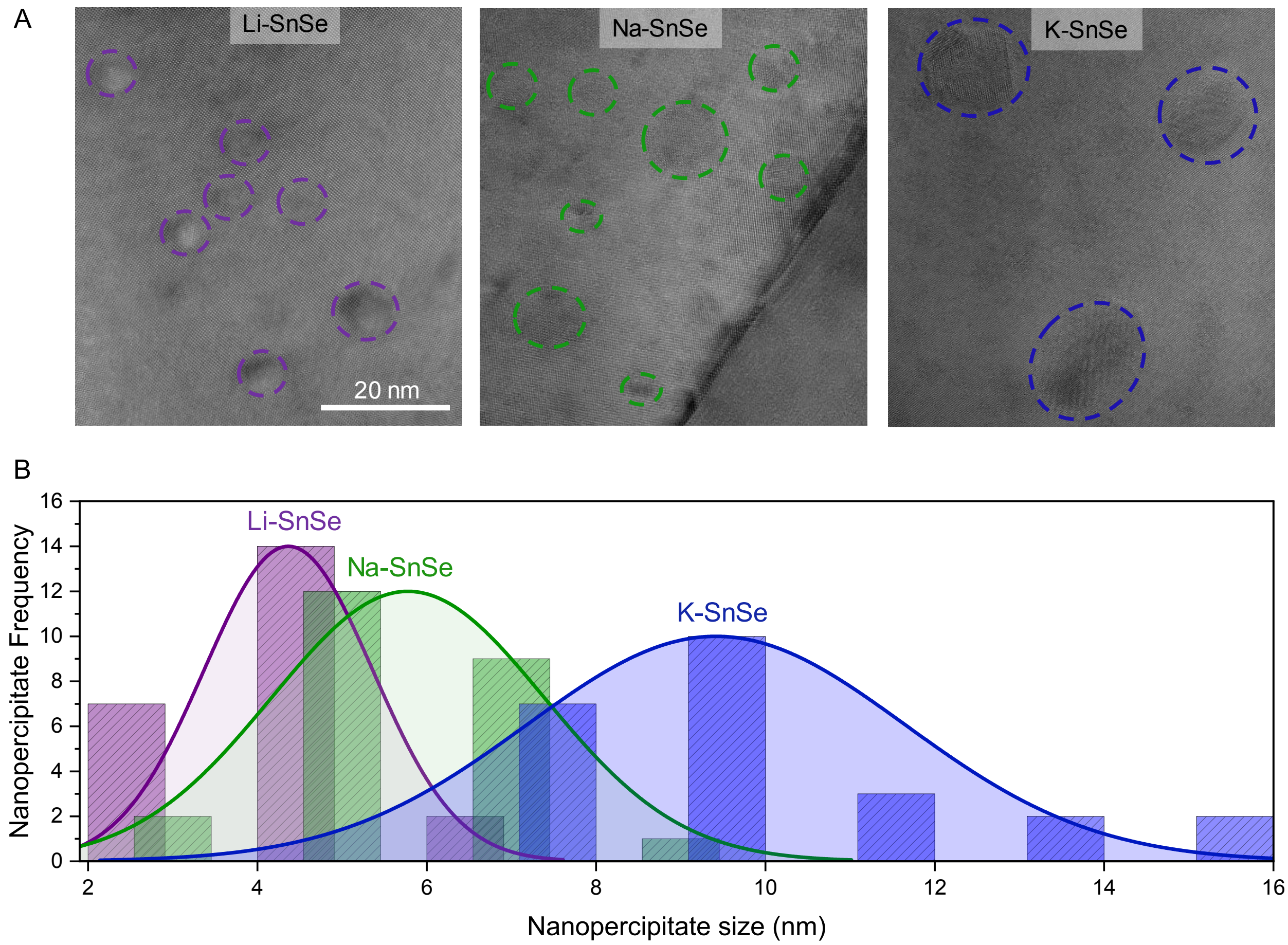


**Figure S11.** Nanoprecipitates present in the different alkali-containing samples: (A) TEM micrographs and (B) size distribution of the nanoprecipitates.

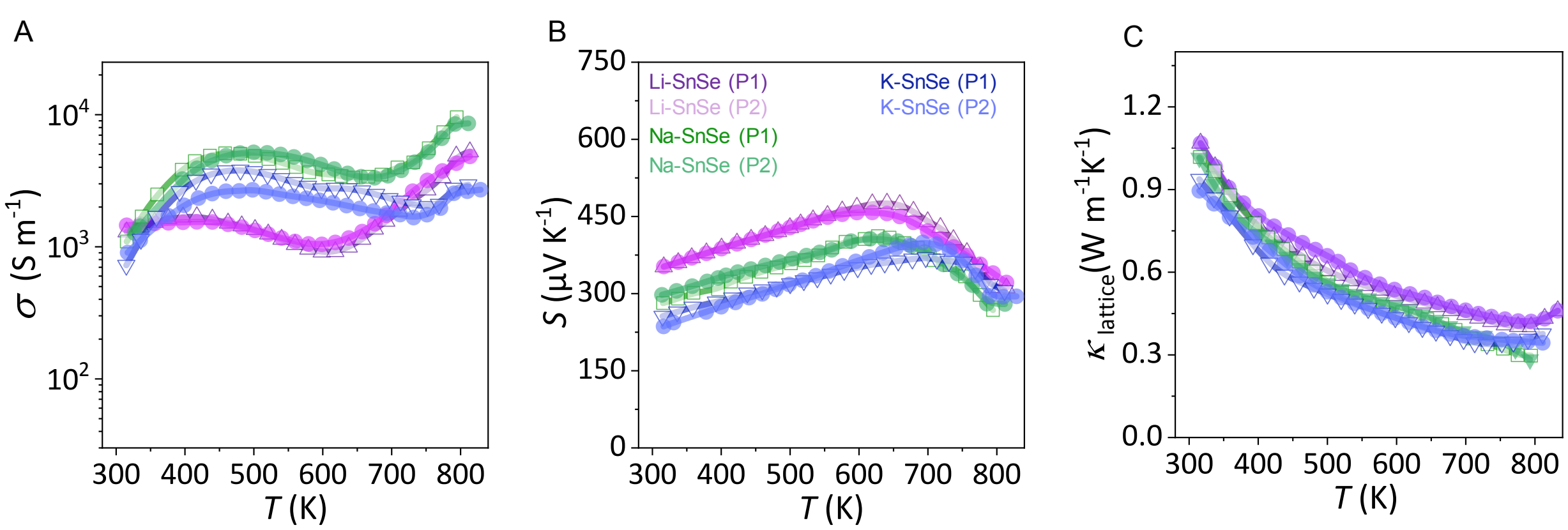

**Figure S12.** Transport properties of the alkali SnSe samples prepared independently by two different operators (P1 and P2) where (A) the electrical conductivity, (B) Seebeck coefficient, (C) lattice thermal conductivity.

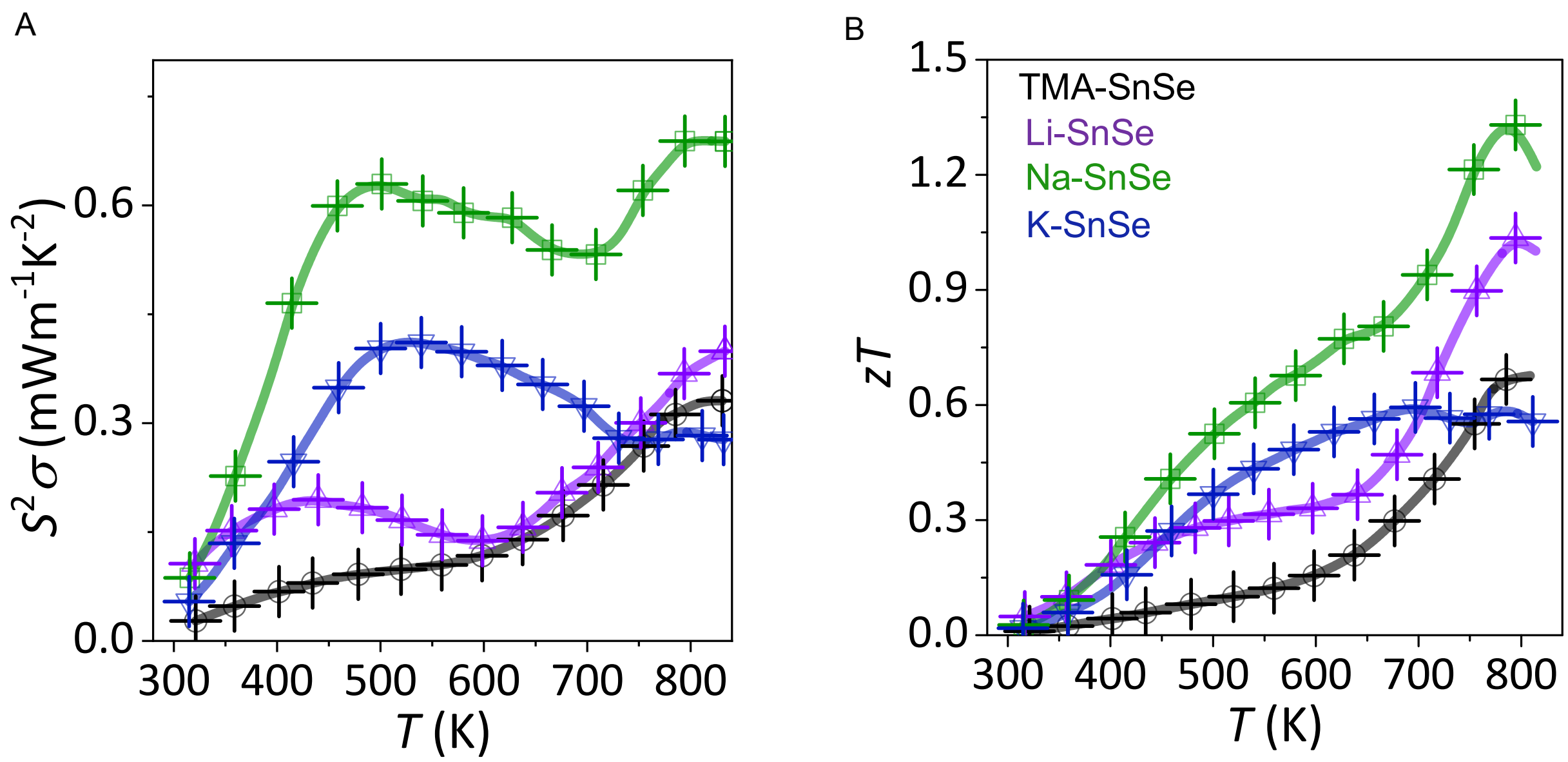


**Figure S13.** Thermoelectric figure of merit ($zT$) of all samples (TMA-SnSe, Li-SnSe, Na-SnSe, and K-SnSe) measured along the direction parallel to the pressing axis.

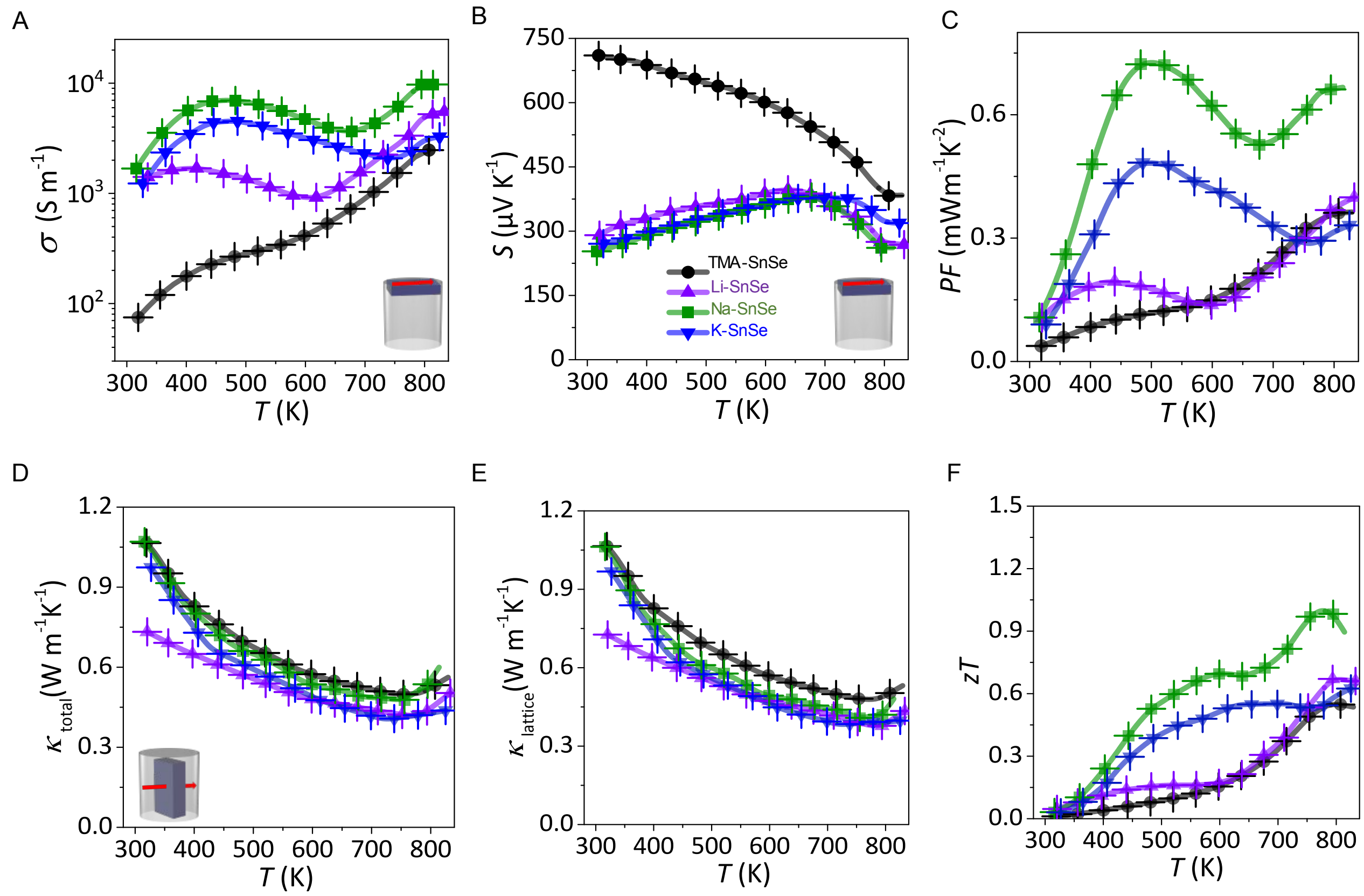


**Figure S14.** Thermoelectric transport properties measured perpendicular to the pressing axis for all SnSe samples (TMA-SnSe, Li-SnSe, Na-SnSe, and K-SnSe): (A) electrical conductivity ($\sigma$), (B) Seebeck coefficient ($S$), (C) power factor ($PF$), (D) total thermal conductivity ($\kappa_{tot}$), (E) lattice thermal conductivity ($\kappa_L$), and (F) thermoelectric figure of merit ($zT$).

## Theoretical *zT* prediction

The single parabolic band (SPB) model was used to calculate the carrier concentration ($p_H$)-dependent $zT$ values (Figure S12) at fixed temperatures of 750 K (dotted lines) and 786 K (solid lines). The lattice thermal conductivity ($\kappa_{lattice}$) used in these calculations was taken from Figure

3E of the main text. The carrier transport properties were evaluated using standard SPB expressions.

The Seebeck coefficient,

$$S(\eta) = \frac{\kappa_B}{e}\left[\frac{(r+5/2)\cdot F_{r+3/2}(\eta)}{(r+3/2)\cdot F_{r+1/2}(\eta)} - \eta\right] \quad \text{(S1)}$$

The Hall carrier concentration,

$$n_H = \frac{1}{e\cdot R_H} = \frac{(2m^*\cdot\kappa_B T)^{3/2}}{3\pi^2\hbar^3}\cdot\frac{(r+3/2\,)^2\cdot F_{r+1/2}^2(\eta)}{(2r+3/2\,)\cdot F_{2r+1/2}(\eta)} \quad \text{(S2)}$$

The Hall mobility,

$$\mu_H = \left[\frac{e\pi\hbar^4}{\sqrt{2}(\kappa_B T)^{3/2}}\frac{C_l}{E_{def}{}^2(m^*)^{5/2}}\right]\frac{(2r+3/2\,)\cdot F_{2r+1/2}(\eta)}{(r+3/2\,)^2\cdot F_{r+1/2}(\eta)} \quad \text{(S3)}$$

Lorenz Factor,

$$L = \left(\frac{\kappa_B}{e}\right)^2\left\{\frac{(r+7/2)\cdot F_{r+5/2}(\eta)}{(r+3/2)\cdot F_{r+1/2}(\eta)} - \left[\frac{(r+5/2)\cdot F_{r+3/2}(\eta)}{(r+3/2)\cdot F_{r+1/2}(\eta)}\right]^2\right\} \quad \text{(S4)}$$

Where,

$$F_x(\eta) = \int_0^\infty \frac{\varepsilon^x}{1+e^{(\varepsilon-\eta)}}\,d\varepsilon \quad \text{(S5)}$$

is the Fermi integral.

$$C_l = \upsilon_l^2\rho \quad \text{(S6)}$$

Here, $S$, $\mu_H$, $\eta$, $\kappa_B$, $e$, $r$, $R_H$, $\hbar$, $C_l$, $E_{def}$, $m^*$, $L$ and $\upsilon_l$ correspond to the Seebeck coefficient, carrier mobility, reduced Fermi level, Boltzmann constant, elementary charge, scattering parameter (with $r$ = -1/2 for acoustic phonon scattering), Hall coefficient, reduced Planck constant, elastic constant

for longitudinal vibrations, deformation potential, density-of-states effective mass, Lorenz number, and longitudinal sound velocity, respectively.[1]

We then compared the experimentally measured *zT* values of the alkali samples with those predicted by the SPB model. Within this framework, the results suggest that the figure of merit could be further improved by increasing the carrier concentration, provided that the carrier mobility is not significantly degraded. Among the alkali ions, Na appears to most closely approach this optimum, as it enables an increase in carrier concentration while largely preserving mobility. In contrast, Li and K fall short of this balance, albeit for different reasons. Lithium tends to distribute more uniformly within the lattice, which limits the formation of beneficial microstructural features and primarily results in conventional doping without additional transport enhancement. Potassium, on the other hand, shows a stronger tendency to segregate, leading to increased disorder and potential disruption of carrier pathways, which can offset gains in carrier concentration. However, the SPB model in SnSe works well up until hole concentrations of *ca*. $2 \times 10^{19}$ $cm^{-3}$. At larger hole concentrations, multi-band transport starts to occur for SnSe and the set of corresponding physics changes similarly. Multi-band transport can enable far superior TE performance than single-band transport and so this is a direction worth pursuing. It should be noted that at higher hole concentrations, SnSe begins to transition into a multi-band transport regime, where the underlying transport physics changes accordingly.[2]

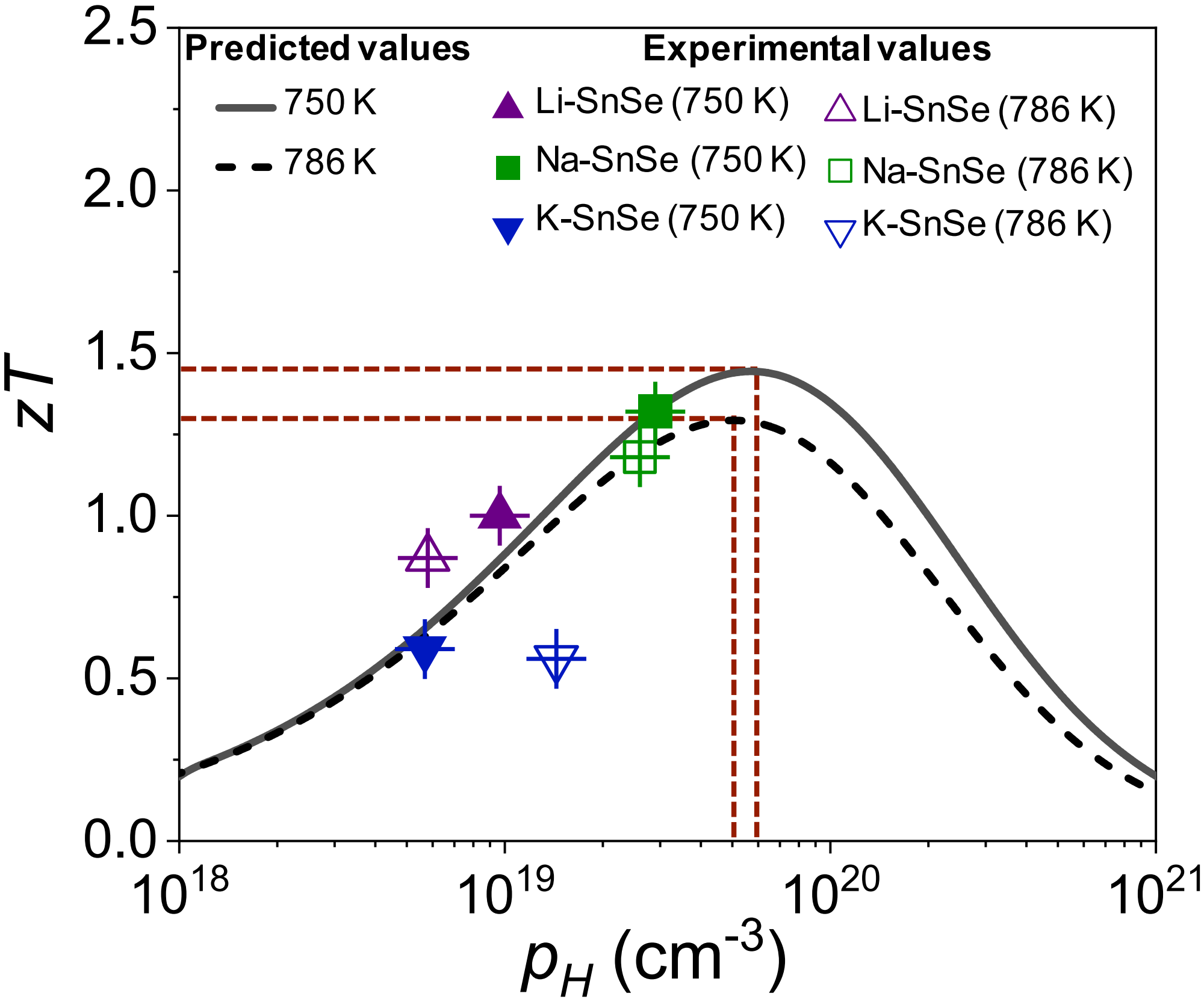


**Figure S15.** Carrier concentration ($p_H$)-dependent $zT$ with both experimental and predicted values at $T$=750 K and 786 K via calculations using the SPB model. The red dashed lines indicate the optimal $p_H$ to achieve peak $zT$ at the given $T$.

**Table S1.** Measured and relative densities of all SnSe pellets made with different reagents obtained from absolute values measured with the Archimedes' method.

| **Sample** | **TMA-SnSe** | **Li-SnSe** | **Na-SnSe** | **K-SnSe** |
|---|---|---|---|---|
| Measured density ($g/cm^3$) | 5.72 | 5.76 | 5.73 | 5.69 |
| Relative density (%) | 92.5 | 93.2 | 92.7 | 92.1 |

**Table S2.** Calculated orientation factors of all samples in-plane and out of plane.

| **Sample** | **Orientation factor (*F*) (⊥)** | **Orientation factor (*F*) (‖)** |
|---|---|---|
| TMA-SnSe | 0.11 | 0.32 |
| Li-SnSe | 0.13 | 0.26 |
| Na-SnSe | 0.15 | 0.28 |
| K-SnSe | 0.13 | 0.41 |

**Table S3.** Literature values of the orientation factors of different SnSe samples reported.

| Sample | Orientation factor (F) (‖) | Orientation factor (F) (⊥) |
|---|---|---|
| $SnSe_{0.92}$ [3] | 0.53 | 0.17 |
| $SnSe_{0.92} + MoCl_5$ [3] | 0.49 | 0.11 |
| SnSe [4] | 0.41 | 0.13 |
| $SnS_{0.2}Se_{0.8}$ [5] | 0.30 | 0.13 |
| $SnSe_{0.95}$ [6] | 0.48 | - |
| $SnSe_{0.95}$ + 0.5%$NbCl_5$ [6] | 0.41 | - |
| $SnSe_{0.95}$-4%$BiCl_3$ [7] | 0.52 | - |
| $SnSe_{0.95}$-3%$PbBr_2$ [8] | 0.50 | - |

**Table S4.** Refinement parameters of synchrotron data of the bulk samples at room temperature ($\lambda$= 0.207377 Å).

| Parameter | TMA-SnSe | Li-SnSe | Na-SnSe | K-SnSe |
|---|---|---|---|---|
| a (Å) | 11.514(7) | 11.504(9) | 11.511(4) | 11.512(1) |
| b (Å) | 4.167(5) | 4.155(2) | 4.159(4) | 4.157(9) |
| c (Å) | 4.421(6) | 4.444(3) | 4.438(2) | 4.441(7) |
| V ($Å^3$) | 212.182(4) | 212.460(8) | 212.493(7) | 212.566(5) |
| $R_p$ | 9.56 | 10.1 | 10.1 | 9.13 |

|  |  |  |  |  |
|---|---|---|---|---|
| $R_{wp}$ | 11.6 | 10.8 | 11.7 | 10.5 |
| $R_{exp}$ | 1.95 | 1.81 | 1.96 | 1.95 |
| $\chi^2$ | 35.28 | 35.6 | 35.2 | 29 |

**Table S5.** Relative apparent density-of-states effective masses estimated from the weighted mobility-to-Hall mobility ratio for Li-SnSe, Na-SnSe, and K-SnSe.

| **Alkali ion** | **μw/μH** | **$(\mu w/\mu H)^{2/3}$ → relative ($m^*/m_e$)** |
|---|---|---|
| Li-SnSe | 2.729 | 1.95 |
| Na-SnSe | 4.52157 | 2.73 |
| K-SnSe | 1.51258 | 1.32 |

**References**

(1) Xiao, Y.; Chang, C.; Pei, Y.; Wu, D.; Peng, K.; Zhou, X.; Gong, S.; He, J.; Zhang, Y.; Zeng, Z.; et al. Origin of low thermal conductivity in SnSe. *Phys. Rev. B* **2016**, *94*.

(2) Chang, C.; Tan, G.; He, J.; Kanatzidis, M. G.; Zhao, L.-D. The Thermoelectric Properties of SnSe Continue to Surprise: Extraordinary Electron and Phonon Transport. *Chem. Mater.* **2018**, *30*, 7355-7367.

(3) Chandra, S.; Bhat, U.; Dutta, P.; Bhardwaj, A.; Datta, R.; Biswas, K. Modular Nanostructures Facilitate Low Thermal Conductivity and Ultra-High Thermoelectric Performance in n-Type SnSe. *Adv. Mater.* **2022**, *34*.

(4) Li, Y.; Shi, X.; Ren, D.; Chen, J.; Chen, L. Investigation of the Anisotropic Thermoelectric Properties of Oriented Polycrystalline SnSe. *Energies* **2015**, *8*, 6275-6285.

(5) Asfandiyar; Wei, T.-R.; Li, Z.; Sun, F.-H.; Pan, Y.; Wu, C.-F.; Farooq, M. U.; Tang, H.; Li, F.; Li, B.; et al. Thermoelectric SnS and SnS-SnSe solid solutions prepared by mechanical alloying and spark plasma sintering: Anisotropic thermoelectric properties. *Sci. Rep.* **2017**, *7*.

(6) Cai, J.; Zhang, Y.; Yin, Y.; Tan, X.; Duan, S.; Liu, G.-Q.; Hu, H.; Xiao, Y.; Ge, Z.; Jiang, J. Investigating the thermoelectric performance of n-type SnSe: the synergistic effect of NbCl5 doping and dislocation engineering. *J. Mater. Chem. C* **2020**, *8*, 13244-13252.

(7) Wang, X.; Xu, J.; Liu, G.; Fu, Y.; Liu, Z.; Tan, X.; Shao, H.; Jiang, H.; Tan, T.; Jiang, J. Optimization of thermoelectric properties in n-type SnSe doped with $BiCl_3$. *App. Phys. Lett.* **2016**, *108*.

(8) Li, D.; Tan, X.; Xu, J.; Liu, G.; Jin, M.; Shao, H.; Huang, H.; Zhang, J.; Jiang, J. Enhanced thermoelectric performance in n-type polycrystalline SnSe by $PbBr_2$ doping. *RSC Adv.* **2017**, *7*, 17906-17912.
